# Triglyceride-glucose-related indices and cardiovascular disease and mortality among individuals with depression: a prospective study from UK Biobank

**DOI:** 10.3389/fnut.2026.1842720

**Published:** 2026-06-23

**Authors:** Shuai Li, Long Xu, Huan Liang, Yuyao Wang, Yueping Shen, Xue Liu

**Affiliations:** 1 Department of Cardiovascular Medicine, Yantai Mountain Hospital, Yantai, Shandong, China; 2 Department of Epidemiology and Biostatistics, School of Public Health, Medical College of Soochow University, Suzhou, Jiangsu, China

**Keywords:** cardiovascular disease, depression, mediation, mortality, predictive performance, triglyceride-glucose indices

## Abstract

**Background:**

The triglyceride-glucose (TyG) index reflects insulin resistance-related metabolic dysfunction and, when combined with anthropometric measures, may capture adiposity-related cardiovascular risk information. Although associated with depression, cardiovascular disease (CVD), and mortality, their associations with incident CVD and mortality among individuals with depression remain unclear. This study examined these associations and explored potential biological correlates.

**Methods:**

This prospective cohort study included 53,171 UK Biobank participants with depression and free of CVD at baseline. Seven TyG-related indices were derived: TyG, TyG-body mass index (TyG-BMI), TyG-waist circumference (TyG-WC), TyG-waist-to-height ratio (TyG-WHtR), TyG-a body shape index (TyG-ABSI), TyG-weight-adjusted waist index (TyG-WWI), and TyG-body roundness index (TyG-BRI). Associations were evaluated using Cox regression models. Incremental predictive information beyond a conventional cardiovascular risk model was assessed using several prediction metrics. Exploratory biomarker analyses identified potential biological correlates.

**Results:**

Over a median follow-up of 13.4 years, 8,516 incident CVD cases, 546 CVD-related deaths, and 4,619 all-cause deaths were documented. For incident CVD, multivariable-adjusted hazard ratios (95% confidence intervals) comparing the highest with the lowest quartile were 1.40 (1.31–1.49) for TyG, 1.80 (1.68–1.92) for TyG-BMI, 1.95 (1.82–2.09) for TyG-WC, 1.89 (1.76–2.03) for TyG-WHtR, 1.77 (1.65–1.90) for TyG-WWI, 1.93 (1.80–2.08) for TyG-BRI, and 1.61 (1.49–1.73) for TyG-ABSI. Each 1-SD increment in these indices was associated with a 24–37% higher risk of CVD mortality and a 7–16% higher risk of all-cause mortality. The associations with incident CVD and CVD mortality showed no significant departure from linearity. Except for TyG and TyG-BMI, adding the other indices to the conventional risk model yielded statistically significant but modest incremental predictive information, with small absolute improvements in prediction metrics. Biomarkers reflecting inflammation (e.g., neutrophils: 6.5–13.1%), metabolism (glycated hemoglobin: 6.5–23.9%), and hepatic (gamma-glutamyltransferase: 5.0–12.3%) and renal (urate: 8.6–25.8%) function statistically accounted for part of their associations with incident CVD.

**Conclusion:**

Higher TyG-related indices were associated with increased risks of incident CVD and mortality among individuals with depression. Several indices provided modest incremental predictive information, but external validation and implementation-focused studies are needed before clinical application. Inflammatory, metabolic, hepatic, and renal biomarkers may represent candidate biological correlates, although causal mediation cannot be inferred.

## 1 Introduction

Cardiovascular disease (CVD) remains the leading cause of mortality worldwide and imposes a substantial global health burden (1). Data from the Global Burden of Disease Study indicate that the number of individuals living with CVD increased from 523 million in 2019 (2) to 612 million in 2021 (3), accounting for 26.8% of all deaths (3). Projections further estimate that CVD-related mortality will escalate to 35.6 million by 2050 (4). Notably, a 2025 European Society of Cardiology consensus statement emphasized the “mental-cardiac connection” as a key clinical issue (5). Depression, one of the most common mental disorders, affects more than 300 million people worldwide (6) and is a well-established risk factor for CVD incidence and mortality (7–10). These observations underscore the need to identify accessible markers that may help characterize cardiovascular risk profiles among individuals with depression.

Metabolic dysregulation may be particularly relevant in this population because depression is associated with multiple biological, behavioral, pharmacological, and healthcare-related cardiovascular risk factors. These include chronic low-grade inflammation, altered autonomic tone, hypothalamic–pituitary–adrenal (HPA) axis dysregulation, oxidative stress, and endothelial dysfunction, reduced physical activity, sleep disturbance, exposure to psychotropic medications, and suboptimal preventive cardiometabolic care (11–17). Depression is also clinically heterogeneous, and recent work on personalized treatment in treatment-resistant depressive disorders has emphasized the importance of multidimensional patient stratification incorporating psychological features such as mentalization, cognitive rigidity, psychache, and suicidality (18). Therefore, simple indices integrating glycemic, lipid, and adiposity-related information may serve as candidate markers for enriching cardiovascular risk assessment in this population.

Insulin resistance (IR), characterized by impaired biological responsiveness to insulin in peripheral tissues (19, 20), contributes to CVD through inflammation, endothelial dysfunction, oxidative stress, and dysregulated lipid metabolism (19–21). IR and depression may also share biological mechanisms, including HPA axis dysregulation, persistent immune activation, and alterations in gut microbiota and neurotransmitter pathways (22, 23). Individuals with depression are at increased risk for IR-related metabolic disorders (22, 23), highlighting the potential relevance of identifying IR-related metabolic dysfunction when characterizing cardiovascular risk in this population. However, direct assessment of IR remains challenging in routine clinical practice. Although the hyperinsulinemic-euglycemic clamp is regarded as the reference standard, its invasiveness, technical complexity, and high cost limit its applicability outside research settings (24). Triglyceride-glucose (TyG)-based indices, including the TyG index and anthropometric derivatives such as TyG-body mass index (TyG-BMI), TyG-waist circumference (TyG-WC), TyG-waist-to-height ratio (TyG-WHtR), TyG-body roundness index (TyG-BRI), TyG-a body shape index (TyG-ABSI), and TyG-weight-adjusted waist index (TyG-WWI), have been proposed as practical indicators of IR-related metabolic dysfunction (25–32). Previous studies have linked TyG-related indices to cardiovascular outcomes in several high-risk populations, including individuals with hypertension (27), diabetes (28), obesity (30), metabolic syndrome (29), metabolic dysfunction-associated steatotic liver disease (31), and cardiovascular-kidney-metabolic syndrome (32). Epidemiological evidence has also shown associations between TyG-related indices and depression (33–35). However, their associations with cardiovascular outcomes and incremental predictive value among individuals with depression remain poorly understood. To date, only two studies have examined TyG-related indices in relation to all-cause mortality among individuals with depression (36, 37), and no previous study has systematically evaluated their associations with incident CVD in this population. Although inflammation, metabolic dysregulation, and multi-organ dysfunction have been proposed as possible biological processes linking TyG-related indices to CVD (27, 30, 38), it is unclear whether similar biomarker patterns are observed among individuals with depression.

Therefore, using data from a large population-based prospective cohort, this study aimed to (1) evaluate the associations between seven TyG-related indices and the risks of incident CVD and mortality among individuals with depression; (2) examine whether adding these indices to a conventional cardiovascular risk model provides incremental predictive information beyond conventional risk factors; and (3) explore possible biological correlates of the associations between TyG-related indices and incident CVD.

## 2 Methods

### 2.1 Data source and study participants

This study was based on data from the UK Biobank, a large and well-characterized prospective cohort comprising more than 500,000 middle-aged and older adults recruited between 2006 and 2010 across England, Scotland, and Wales. At baseline, participants completed detailed questionnaires, underwent standardized physical examinations, and provided biological specimens. Comprehensive descriptions of the cohort design and data collection procedures have been published previously (39). Ethical approval was granted by the North West Multi-Centre Research Ethics Committee, and all participants provided written informed consent.

**Figure 1:**
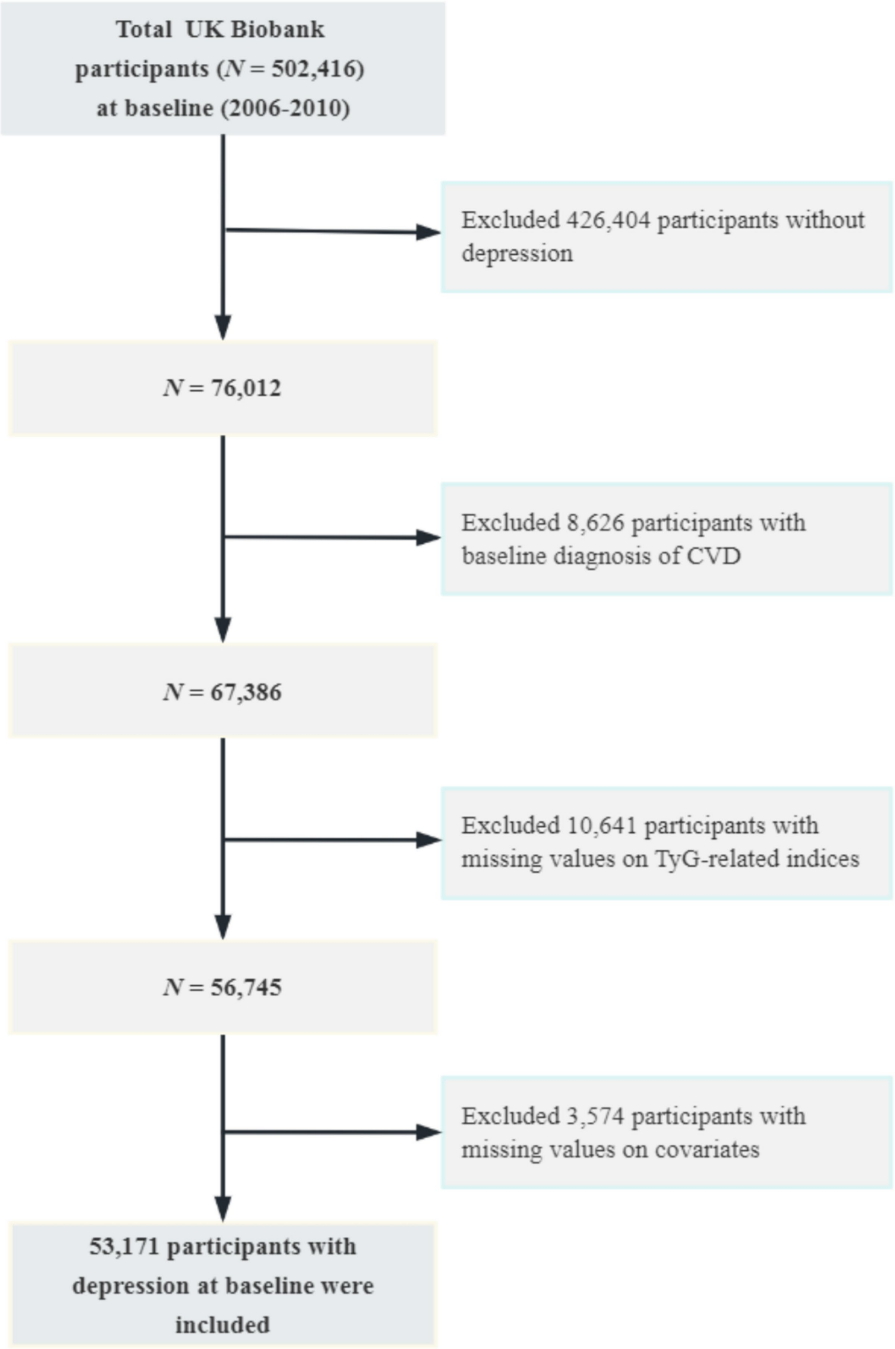
Flowchart of participant selection.

Consistent with prior UK Biobank studies (40, 41), baseline depression was defined using an integrated definition based on four data sources: depressive symptoms assessed using the Patient Health Questionnaire-2 (PHQ-2), self-reported physician diagnoses, antidepressant prescription records, and hospital admission records with International Classification of Diseases, 10th Revision (ICD-10) codes F32–F33 (Supplementary Table S1). PHQ-2 scores range from 0 to 6, and a cutoff value ≥3 is commonly applied to indicate probable depression (7). This integrated definition was used to improve case ascertainment by capturing symptom-based, self-reported, treatment-based, or hospital-record-based evidence of depression. These sources may reflect different aspects of depression, including current depressive symptoms, previous physician-diagnosed depression, treated depression, and more clinically severe depression requiring hospital-based care. Thus, this definition was intended to identify a broad cohort of participants with depression at baseline rather than a clinically homogeneous depression phenotype. Participants were classified as having baseline depression if they met at least one of these criteria. Because the criteria were not mutually exclusive, participants could meet more than one criterion but were counted only once in the analytic cohort. Using this integrated definition, 76,012 participants were identified as having depression at baseline. Participants with prevalent CVD at baseline (*n* = 8,626), missing TyG-related indices (*n* = 10,641), or incomplete covariate information (*n* = 3,574) were then excluded sequentially. TyG-related indices were not imputed because they were the primary exposures. To assess the potential influence of excluding participants with missing TyG-related indices, baseline characteristics were compared between participants with complete versus missing TyG-related index data in the Supplementary material. The final analytic sample included 53,171 participants with depression who were free of CVD at baseline (Figure 1).

### 2.2 TyG-related indices

Peripheral venous blood was obtained by the UK Biobank from randomly selected participants following the provision of written informed consent. After fractionation and rigorous quality-control procedures, biospecimens were stored at −80 °C and subsequently used to quantify multiple biochemical markers, including triglycerides (TG), glucose, and high-density lipoprotein cholesterol (HDL-C). All assays were conducted in a central laboratory using a Beckman Coulter AU5800 automated analyzer, with coefficients of variation <3% for both TG and glucose (42). Detailed protocols have been reported elsewhere (39, 42). Baseline anthropometric assessments, including height, weight, and WC, were performed. Seven TyG-related indices were calculated, comprising the original TyG index; three traditional composite measures (TyG-BMI, TyG-WC, and TyG-WHtR); and three novel composite indices (TyG-BRI, TyG-ABSI, and TyG-WWI). Because TG and glucose concentrations in the UK Biobank data are recorded in mmol/L, values were converted to mg/dL using standard conversion factors prior to calculating these indices (25). The formulas used to calculate the seven TyG-related indices are presented in Equations 1–7. Formula definitions and measurement units for each TyG-derived index were based on previously published sources (31, 32).

TyGindex=ln[TG(mg/dL)×glucose(mg/dL)/2]; (1)

$$\mathrm{TyG}-\mathrm{BMI}\;\text{index}=\mathrm{TyG}\times \frac{\text{weight}(\mathrm{kg})}{\text{height}{\left(m\right)}^{2}};$$ (2)

TyG−WCindex=TyG×WC(cm); (3)

$$\mathrm{TyG}-\text{WHtR index}=\mathrm{TyG}\times \frac{\mathrm{WC}(\mathrm{cm})}{\text{height}(\mathrm{cm})};$$ (4)

$$\mathrm{TyG}-\text{ABSI index}=\mathrm{TyG}\times \frac{\mathrm{WC}(m)}{\mathrm{BM}I^{2/3}\times \text{height}{\left(m\right)}^{1/2}};$$ (5)

$$\mathrm{TyG}-\mathrm{WWI}\;\text{index}=\mathrm{TyG}\times \frac{\mathrm{WC}(\mathrm{cm})}{\sqrt{\text{weight}(\mathrm{kg})}};$$ (6)

$$\begin{array}{l} \mathrm{TyG}-\mathrm{BRI}\;\text{index}=\mathrm{TyG}\\ \times [364.2-365.5\times \sqrt{1-{\left(\mathrm{WC}(m)/2\pi \right)}^{2}/{\left(\text{height}(m)/2\right)}^{2}}]. \end{array}$$ (7)

### 2.3 Candidate intermediary biomarkers

Building on prior findings regarding plausible biological processes (27, 30, 38), we incorporated a broad panel of baseline biomarkers to explore their potential contributions to the observed associations between TyG-related indices and incident CVD. Biomarkers were classified into four functional domains: (1) liver-related measures (albumin, alanine aminotransferase [ALT], aspartate aminotransferase [AST], alkaline phosphatase [ALP], direct bilirubin, gamma-glutamyltransferase [GGT], total bilirubin [TB], and total protein [TP]); (2) kidney-related measures (urea, creatinine, cystatin C, phosphate, and urate); (3) systemic inflammation indicators (C-reactive protein [CRP], total white blood cell count [WBC], lymphocyte, monocyte, neutrophil, eosinophil, and basophil counts, platelet count, and platelet crit); and (4) metabolic markers (apolipoprotein A, apolipoprotein B, total cholesterol, glycated hemoglobin [HbA1c], HDL-C, LDL-C, and lipoprotein(a); Supplementary Table S2).

### 2.4 Study outcomes

This study examined three pre-specified primary outcomes: incident CVD, CVD mortality, and all-cause mortality. Incident events were identified through linkage to national inpatient hospital records, including Hospital Episode Statistics for England, Scottish Morbidity Records for Scotland, and the Patient Episode Database for Wales (39). Mortality data, including date of death and certified underlying cause, were obtained from national death registries maintained by the NHS Information Centre for England and Wales and the NHS Central Register for Scotland (39). Incident CVD events were defined as the first occurrence after baseline of coronary heart disease (CHD) (ICD-10 codes: I20–I25), atrial fibrillation (AF) (I48), heart failure (HF) (I50), or stroke (I60–I64). CVD mortality was defined as death with an underlying cause coded as CHD, AF, HF, or stroke. All-cause mortality was defined as death from any cause. The composite incident CVD outcome was used to capture the overall burden of major cardiovascular events among individuals with depression and to maintain comparability with prior UK Biobank studies using broad CVD definitions (43, 44). This approach also provided greater statistical power for evaluating the overall cardiovascular risk associated with TyG-related indices. However, CHD, AF, HF, and stroke are clinically and biologically heterogeneous outcomes. Therefore, the composite CVD endpoint should be interpreted as an overall cardiovascular burden measure rather than as a single disease entity with uniform pathophysiological characteristics. We also examined each CVD subtype, including CHD, AF, HF, and stroke, as secondary outcomes. Participants without CVD at baseline were followed prospectively from the initial assessment visit until the first occurrence of a CVD event, death, or censoring at the end of follow-up (February 27, 2022), whichever came first.

### 2.5 Covariates

Covariates were defined using baseline characteristics. The primary adjustment set included demographic, socioeconomic, and lifestyle factors that were considered potential upstream common causes of both TyG-related indices and cardiovascular outcomes. Demographic variables included age (years, continuous), sex, and race (White vs. non-White). Socioeconomic factors included the Townsend Deprivation Index (TDI; continuous), employment status (employed vs. unemployed), and educational attainment (college degree or higher vs. lower). Lifestyle factors included smoking status (never, former, or current), drinking frequency (never, special occasions only, 1–3 times/month, 1–2 times/week, 3–4 times/week, or daily/almost daily), physical activity (PA; adequate vs. inadequate), and sleep duration (7–8 h/day vs. <7 or >8 h/day). The TDI is used as a measure of area-level socioeconomic deprivation, with higher values indicating greater deprivation (45). Adequate PA was defined as ≥75 min/week of vigorous-intensity activity, ≥150 min/week of moderate-intensity activity, or an equivalent combination of both (46). The primary covariate adjustment strategy was based on a directed-acyclic-graph framework (Supplementary Figure S1). The primary models aimed to estimate the total association between TyG-related indices and cardiovascular outcomes. Therefore, demographic, socioeconomic, and lifestyle factors were included as upstream confounders. In contrast, hypertension, diabetes, and dyslipidemia were not included in the primary adjustment set because they may occur downstream of TyG-related metabolic dysfunction and adiposity and may therefore represent intermediate cardiometabolic states on the pathway from TyG-related indices to CVD (47). Adjustment for these variables in the main model could attenuate the total association through overadjustment. Nevertheless, these variables may also be interpreted as potential confounders in some causal frameworks, particularly if these conditions predate the baseline measurement of TyG-related indices. Participants with missing values for any covariates were excluded before the primary analysis because the proportion of missing data was low (maximum 3.3%; Supplementary Table S3). Detailed definitions of covariates are provided in Supplementary Table S4.

### 2.6 Statistical analysis

Baseline characteristics were summarized using appropriate descriptive statistics and were presented according to quartiles of the TyG index (Q1–Q4) in the main analysis. Baseline characteristics stratified by incident CVD status were additionally provided in the Supplementary material. Normality of continuous variables was assessed using the Shapiro–Wilk test. Normally distributed variables were presented as means ± standard deviation (SD), non-normally distributed variables as medians (interquartile range, IQR), and categorical variables as frequencies and proportions. Between-group differences were evaluated using Student’s *t*-test, Kruskal–Wallis test, or chi-square test, as appropriate.

Associations between TyG-related indices and study outcomes were examined using Cox proportional hazards models with follow-up time as the underlying timescale, after confirming the proportional hazards assumption using Schoenfeld residuals. Restricted cubic spline (RCS) functions were applied to assess potential nonlinear dose–response relationships, with the optimal number of knots selected by minimizing the Akaike Information Criterion (AIC) (48). Effect estimates were reported as hazard ratios (HRs) with 95% confidence intervals (CIs) for TyG-related indices analyzed both continuously (per SD increase) and categorically (quartiles). Because TyG-related indices share common components and are expected to be correlated, each TyG-related index was evaluated in a separate model rather than being included simultaneously. Therefore, comparisons across indices should be interpreted as descriptive rather than as mutually adjusted or definitive rankings. RCS analyses were used not only to assess potential nonlinearity but also to guide the choice of reference groups in quartile-based analyses. For outcomes showing approximately monotonic associations, the lowest quartile was used as the reference group. For outcomes showing nonlinear patterns, the quartile corresponding to the lowest observed risk on the spline curve was selected as the reference group. Because reference categories may differ across outcomes, quartile-based HRs should be interpreted relative to the outcome-specific reference group rather than as direct cross-outcome comparisons. All models were adjusted for age, sex, race, employment status, educational attainment, TDI, smoking status, alcohol consumption frequency, sleep duration, and PA. Kaplan–Meier (KM) curves were generated according to quartiles of TyG-related indices, and differences were assessed using log-rank tests.

To examine whether TyG-related indices provided incremental predictive information beyond conventional cardiovascular risk factors, model performance was evaluated using complementary measures of discrimination, reclassification, and calibration, consistent with established prediction-model assessment frameworks (49, 50). We computed the concordance index (C-index), net reclassification improvement (NRI), and integrated discrimination improvement (IDI) (31). The C-index was used to assess model discrimination, whereas NRI and IDI were used to evaluate changes in risk classification after adding each TyG-related index to the conventional model (49). Each TyG-related index was added to the conventional model separately. Thus, the analyses were designed to estimate the incremental information provided by each index individually rather than to compare their mutually independent effects. The conventional model was constructed using variables from the Framingham General Cardiovascular Risk Score (51), including age, sex, smoking status, systolic blood pressure (SBP), antihypertensive medication use, diabetes, total cholesterol, and HDL-C. To assess model calibration, bootstrap-corrected calibration plots were generated at the 10-year time horizon for incident CVD (50). Predicted 10-year incident CVD risks were compared with KM-observed risks, and calibration curves were generated for the conventional model and for models additionally including each TyG-related index. Consistent with previous studies (31, 52, 53), these analyses were intended to assess the incremental predictive contribution of each TyG-related index. They were not designed to establish clinical utility, determine readiness for routine implementation, or demonstrate that TyG-related composite indices outperform their individual anthropometric components or conventional risk factors.

Exploratory biomarker-based mediation analyses were conducted to identify candidate intermediary biomarkers that may statistically account for part of the associations between TyG-related indices and incident CVD. In accordance with prior frameworks (30, 54, 55), candidate biomarkers were selected using a two-step approach: multivariable-adjusted linear regression to assess cross-sectional associations between TyG-related indices and baseline biomarkers, followed by covariate-adjusted Cox models to evaluate prospective associations between baseline biomarkers and CVD incidence. Biomarkers showing statistically significant and directionally consistent associations in both steps were included in mediation models. Mediation analyses were conducted using the ‘*mediation’* package in R, with the mediating proportion estimated and corresponding 95% CIs derived from 1,000 nonparametric bootstrap resamples. All biomarkers were standardized prior to analysis. Because TyG-related indices and biomarkers were measured at the same baseline assessment, these analyses were considered exploratory and hypothesis-generating rather than confirmatory causal mediation analyses.

Several sensitivity analyses were conducted to assess the robustness of the findings. First, to minimize potential reverse causation, we excluded events occurring within the first 2 years of follow-up. Second, we repeated the analyses after multiple imputation for covariates with missing values. Third, because hypertension and diabetes may also be interpreted as confounders if they predate baseline TyG-related indices, additional sensitivity analyses were conducted with further adjustment for baseline hypertension and diabetes. Fourth, given the potential heterogeneity introduced by the integrated depression definition, we conducted additional sensitivity analyses using stricter definitions of depression, including analyses restricted to participants with ICD-10-coded depression and to those with PHQ-2-defined probable depression. Fifth, to account for competing risks, Fine–Gray subdistribution hazard models were fitted for incident CVD and CVD mortality, with non-CVD death treated as a competing event. Finally, subgroup analyses were performed stratified by sociodemographic and lifestyle factors. All analyses were conducted using R version 4.4.0. Statistical significance was defined as a two-sided *p* < 0.05, with false discovery rate (FDR)-adjusted *p* < 0.05 applied to biomarker-related analyses.

## 3 Results

### 3.1 Baseline characteristics of study participants

Among 53,171 participants with depression, the median age was 56 years, and 65.7% were female. Baseline characteristics according to quartiles of the TyG index are presented in Table 1. Participants with higher levels of the TyG index were generally older, more likely to be male, and had less favorable socioeconomic and lifestyle profiles (all *p* < 0.001). Similar patterns were observed when baseline characteristics were stratified according to incident CVD status (Supplementary Table S5).

**Table 1:** Baseline characteristics of study population according to the quartiles of the TyG index.

Characteristics	TyG index				*P*-values
	Q1	Q2	Q3	Q4	
*n*	13,280	13,310	13,281	13,300	
Age (years)	52.00 (46.00, 59.00)	56.00 (49.00, 62.00)	57.00 (50.00, 62.00)	57.00 (50.00, 62.00)	<0.001
Sex					<0.001
Male	3,133 (23.6)	3,860 (29.0)	4,872 (36.7)	6,367 (47.9)	
Female	10,147 (76.4)	9,450 (71.0)	8,409 (63.3)	6,933 (52.1)	
Ethnicity					<0.001
White	12,252 (92.3)	12,525 (94.1)	12,578 (94.7)	12,585 (94.6)	
Non-white	1,028 (7.7)	785 (5.9)	703 (5.3)	715 (5.4)	
Employed status					<0.001
Employed	11,368 (85.6)	11,313 (85.0)	11,179 (84.2)	10,672 (80.2)	
Not employed	1,912 (14.4)	1,997 (15.0)	2,102 (15.8)	2,628 (19.8)	
Educational level					<0.001
University or college	4,783 (36.0)	4,046 (30.4)	3,695 (27.8)	3,464 (26.0)	
Others	8,497 (64.0)	9,264 (69.6)	9,586 (72.2)	9,836 (74.0)	
TDI	−1.76 (−3.49, 1.21)	−1.87 (−3.47, 1.13)	−1.74 (−3.44, 1.32)	−1.38 (−3.24, 1.75)	<0.001
Physical activity					<0.001
Adequate	8,082 (60.9)	7,712 (57.9)	7,288 (54.9)	6,759 (50.8)	
Inadequate	5,198 (39.1)	5,598 (42.1)	5,993 (45.1)	6,541 (49.2)	
Smoking status					<0.001
Never smoking	7,430 (55.9)	6,958 (52.3)	6,614 (49.8)	6,189 (46.5)	
Ever smoking	4,079 (30.7)	4,439 (33.4)	4,606 (34.7)	4,718 (35.5)	
Current smoking	1,771 (13.3)	1,913 (14.4)	2,061 (15.5)	2,393 (18.0)	
Drinking frequency					<0.001
Never	1,368 (10.3)	1,572 (11.8)	1,632 (12.3)	1,745 (13.1)	
Special occasions only	1,741 (13.1)	1,979 (14.9)	2,087 (15.7)	2,269 (17.1)	
1–3 times/month	1,572 (11.8)	1,672 (12.6)	1,699 (12.8)	1,694 (12.7)	
1–2 times/week	3,251 (24.5)	3,201 (24.0)	3,118 (23.5)	3,080 (23.2)	
3–4 times/week	2,697 (20.3)	2,485 (18.7)	2,395 (18.0)	2,196 (16.5)	
Daily or almost daily	2,651 (20.0)	2,401 (18.0)	2,350 (17.7)	2,316 (17.4)	
Sleep duration					<0.001
7–8 h/day	9,171 (69.1)	8,956 (67.3)	8,860 (66.7)	8,449 (63.5)	
<7 or >8 h/day	4,109 (30.9)	4,354 (32.7)	4,421 (33.3)	4,851 (36.5)	
TyG index	8.10 (7.91, 8.23)	8.54 (8.44, 8.63)	8.91 (8.81, 9.01)	9.43 (9.26, 9.68)	<0.001
TyG-BMI index	199.03 (179.31, 223.86)	226.97 (205.09, 254.96)	250.10 (224.58, 280.43)	281.30 (252.61, 316.76)	<0.001
TyG-WC index	650.05 (589.27, 726.54)	744.49 (674.37, 824.16)	822.96 (749.36, 905.05)	929.18 (849.95, 1019.86)	<0.001
TyG-WHtR index	3.92 (3.57, 4.34)	4.47 (4.09, 4.91)	4.92 (4.51, 5.38)	5.51 (5.06, 6.04)	<0.001
TyG-WWI index	78.99 (74.33, 83.81)	86.54 (82.13, 91.19)	92.76 (88.26, 97.33)	101.18 (96.03, 106.62)	
TyG-ABSI index	0.59 (0.56, 0.63)	0.64 (0.61, 0.68)	0.69 (0.65, 0.72)	0.75 (0.71, 0.78)	
TyG-BRI index	8.10 (7.91, 8.23)	8.54 (8.44, 8.63)	8.91 (8.81, 9.01)	9.43 (9.26, 9.68)	

Data are presented as median (interquartile range) for continuous variables and *n* (%) for categorical variables.

CVD, cardiovascular disease; TDI, Townsend deprivation index; TyG, triglyceride-glucose index; BMI, body mass index; WC, waist circumference; WHtR, weight-to-height ratio; BRI, body roundness index; ABSI, a body shape index; WWI, weight-adjusted waist index.

### 3.2 Associations between TyG-related indices and incident CVD

During a median follow-up of 13.4 years (IQR: 12.5–14.2), 8,516 incident CVD events occurred. Higher TyG index levels were associated with increased CVD risk (log-rank *p* < 0.001; Supplementary Figure S2A). RCS analyses showed no significant departure from linearity for this association (*P* for non-linearity = 0.990; Supplementary Figure S2D). Each SD increment in the TyG index was associated with a 14% higher risk of incident CVD (HR = 1.14, 95% CI: 1.11–1.16), and participants in the highest TyG quartile had a significantly higher risk than those in the lowest quartile (HR = 1.40; 95% CI: 1.31–1.49) (Table 2).

**Table 2:** Associations between TyG-related indices and incident total CVD and mortality in participants with depression.

Exposures	Total CVD incidence		All-cause mortality		CVD mortality	
	Cases/Person-years	HR (95% CI)	Cases/Person-years	HR (95% CI)	Cases/Person-years	HR (95% CI)
TyG index						
Per SD increment		1.14 (1.11–1.16)		1.03 (1.01–1.06)		1.18 (1.09–1.29)
Quartile 1	1,427/170,367	Reference	870/179,335	1.08 (0.99–1.18)	70/179,335	Reference
Quartile 2	1,980/166,825	1.14 (1.07–1.23)	1,053/178,849	Reference	118/178,849	1.26 (0.94–1.69)
Quartile 3	2,339/163,312	1.25 (1.17–1.34)	1,247/177,255	1.06 (0.98–1.15)	156/177,255	1.46 (1.10–1.94)
Quartile 4	2,770/160,603	1.40 (1.31–1.49)	1,449/177,049	1.12 (1.03–1.21)	202/177,049	1.64 (1.24–2.16)
TyG-BMI index						
Per SD increment		1.28 (1.25–1.31)		1.07 (1.04–1.11)		1.24 (1.14–1.35)
Quartile 1	1,361/170,130	Reference	997/178,589	1.19 (1.09–1.29)	83/178,589	Reference
Quartile 2	1,911/166,857	1.18 (1.10–1.27)	1,046/178,369	Reference	122/178,369	1.12 (0.85–1.48)
Quartile 3	2,325/164,212	1.38 (1.29–1.47)	1,164/178,215	1.02 (0.94–1.11)	148/178,215	1.21 (0.92–1.59)
Quartile 4	2,919/159,907	1.80 (1.68–1.92)	1,412/177,315	1.23 (1.13–1.33)	193/177,315	1.59 (1.23–2.07)
TyG-WC index						
Per SD increment		1.32 (1.29–1.35)		1.13 (1.10–1.17)		1.33 (1.22–1.46)
Quartile 1	1,195/172,099	Reference	781/179,943	1.04 (0.94–1.14)	65/179,943	Reference
Quartile 2	1,767/168,183	1.23 (1.14–1.33)	973/178,837	Reference	93/178,837	1.02 (0.74–1.40)
Quartile 3	2,362/163,479	1.50 (1.40–1.61)	1,232/177,746	1.08 (0.99–1.17)	159/177,746	1.37 (1.02–1.85)
Quartile 4	3,192/157,345	1.95 (1.82–2.09)	1,633/175,961	1.26 (1.16–1.37)	229/175,961	1.66 (1.23–2.23)
TyG-WHtR index						
Per SD increment		1.30 (1.27–1.33)		1.13 (1.10–1.17)		1.35 (1.24–1.47)
Quartile 1	1,202/171,822	Reference	788/179,680	1.04 (0.95–1.14)	70/179,680	Reference
Quartile 2	1,807/167,813	1.21 (1.13–1.30)	1,009/178,691	Reference	100/178,691	0.99 (0.73–1.35)
Quartile 3	2,355/163,786	1.44 (1.34–1.54)	1,198/177,910	1.01 (0.93–1.10)	135/177,910	1.08 (0.80–1.45)
Quartile 4	3,152/157,685	1.89 (1.76–2.03)	1,624/176,206	1.25 (1.16–1.36)	241/176,206.	1.75 (1.33–2.30)
TyG-WWI index						
Per SD increment		1.23 (1.21–1.26)		1.16 (1.12–1.19)		1.37 (1.26–1.50)
Quartile 1	1,158/172,949	Reference	657/180,563	Reference	54/180,563	Reference
Quartile 2	1,854/167,476	1.29 (1.20–1.39)	983/178,882	1.10 (0.99–1.21)	95/178,882	1.19 (0.85–1.66)
Quartile 3	2,350/163,544	1.44 (1.34–1.55)	1,234/177,629	1.14 (1.04–1.26)	143/177,629	1.40 (1.02–1.93)
Quartile 4	3,154/157,138	1.77 (1.65–1.90)	1,745/175,414	1.37 (1.24–1.50)	254/175,414	2.01 (1.48–2.74)
TyG-BRI index						
Per SD increment		1.28 (1.26–1.31)		1.15 (1.11–1.18)		1.33 (1.23–1.44)
Quartile 1	1,209/171,717	Reference	807/179,577	1.04 (0.95–1.15)	70/179,577	Reference
Quartile 2	1,795/167,669	1.22 (1.14–1.32)	998/178,658	Reference	104/178,658	1.05 (0.78–1.43)
Quartile 3	2,364/164,117	1.46 (1.36–1.56)	1,175/178,179	0.99 (0.91–1.08)	145/178,179	1.17 (0.87–1.57)
Quartile 4	3,148/157,603	1.93 (1.80–2.07)	1,639/176,074	1.28 (1.18–1.39)	227/176,074	1.69 (1.28–2.24)
TyG-ABSI index						
Per SD increment		1.19 (1.16–1.22)		1.16 (1.13–1.20)		1.34 (1.22–1.47)
Quartile 1	1,196/171,451	Reference	626/179,378	Reference	45/179,378	Reference
Quartile 2	1,873/168,728	1.25 (1.16–1.34)	983/180,102	1.14 (1.03–1.27)	105/180,102	1.55 (1.09–2.21)
Quartile 3	2,336/163,070	1.37 (1.27–1.47)	1,233/177,141	1.20 (1.09–1.33)	134/177,141	1.54 (1.09–2.18)
Quartile 4	3,111/157,858	1.61 (1.49–1.73)	1,777/175,866	1.43 (1.29–1.58)	262/175,866	2.30 (1.63–3.23)

Reference quartiles for categorical analyses were selected according to the dose–response patterns observed in restricted cubic spline analyses. For approximately monotonic associations, Q1 was used as the reference group. For nonlinear associations, the quartile corresponding to the lowest observed risk was selected as the reference group. Models were adjusted for age, sex, race, employment status, educational level, Townsend deprivation index, smoking status, drinking frequency, sleep duration, and physical activity.

CVD, cardiovascular disease; SD, standard deviation; HR, hazard ratio; CI, confidence interval; TyG, triglyceride-glucose; BMI, body mass index; WC, waist circumference; WHtR, waist-to-height ratio; BRI, body roundness index; ABSI, a body shape index; WWI, weight-adjusted waist index.

**Figure 2:**
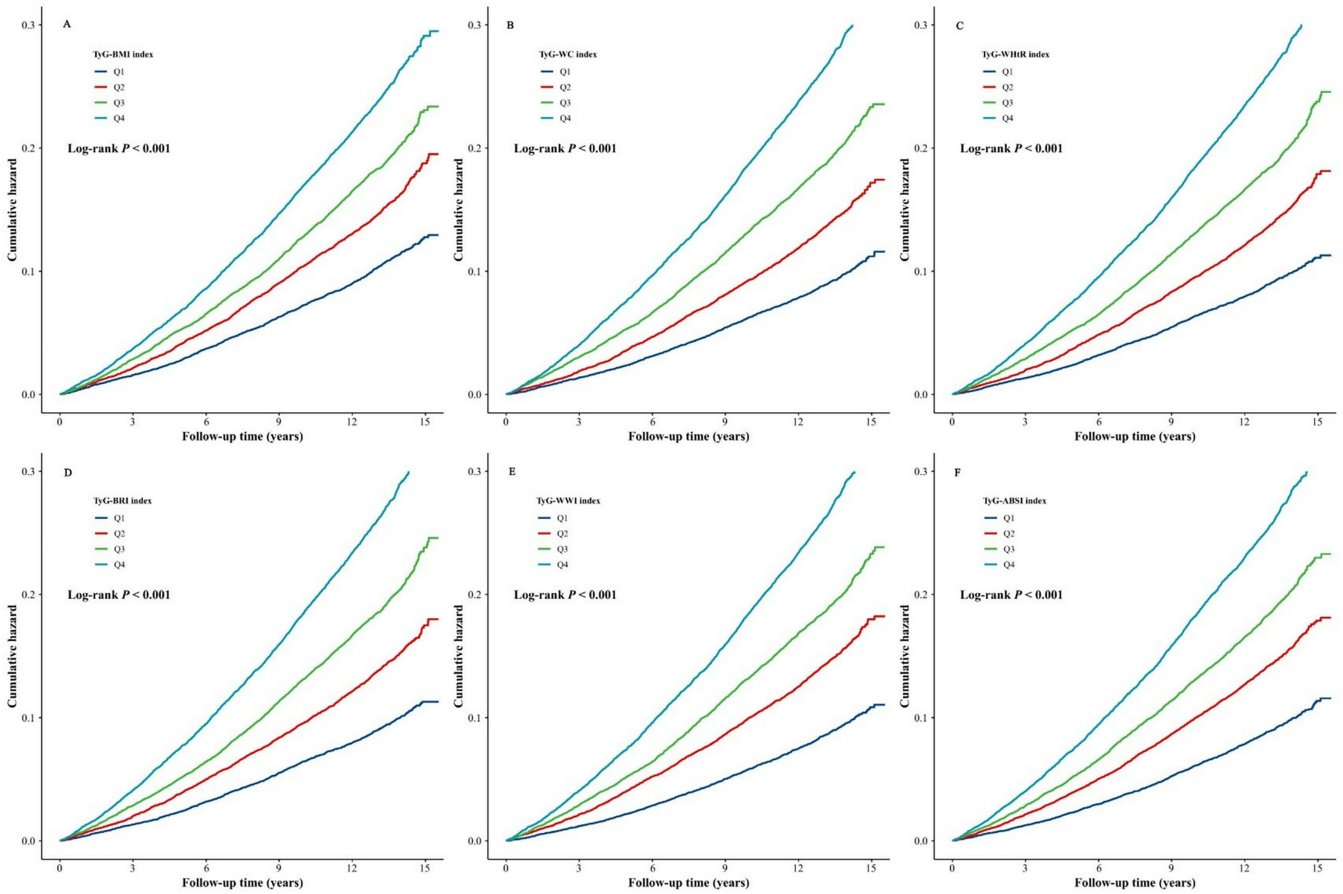
Kaplan–Meier curves of incident CVD according to the quartiles of TyG-related indices in participants with depression. **(A)** TyG-BMI index; **(B)** TyG-WC index; **(C)** TyG-WHtR index; **(D)** TyG-BRI index; **(E)** TyG-WWI index; and **(F)** TyG-ABSI index. Q, Quartile; HR, hazard ratio; CI, confidence interval; CVD, cardiovascular disease; TyG, triglyceride-glucose; BMI, body mass index; WC, waist circumference; WHtR, waist-to-height ratio; BRI, body roundness index; ABSI, a body shape index; WWI, weight-adjusted waist index.

**Figure 3:**
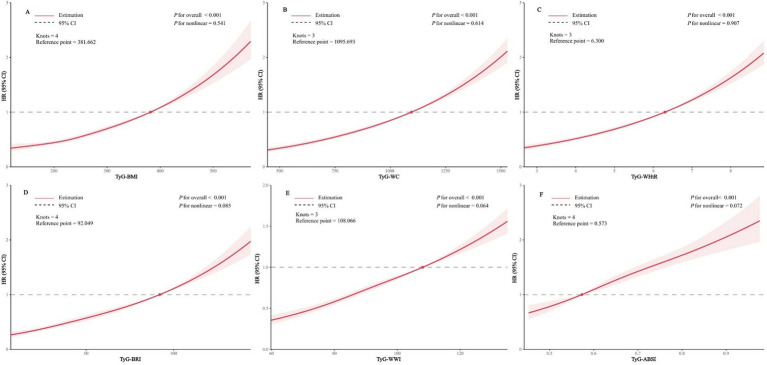
Dose–response relationship of TyG-related indices with incident CVD by RCS analysis in participants with depression. RCS analysis was derived from Cox proportional hazards models adjusted for age, sex, race, employment status, educational level, Townsend deprivation index, smoking status, drinking frequency, sleep duration, and physical activity. **(A)** TyG-BMI index; **(B)** TyG-WC index; **(C)** TyG-WHtR index; **(D)** TyG-BRI index; **(E)** TyG-WWI index; and **(F)** TyG-ABSI index. HR, hazard ratio; CI, confidence interval; CVD, cardiovascular disease; RCS, restricted cubic spline; TyG, triglyceride-glucose; BMI, body mass index; WC, waist circumference; WHtR, waist-to-height ratio; BRI, body roundness index; ABSI, a body shape index; WWI, weight-adjusted waist index.

Similar positive associations were observed for the other TyG-related indices. Participants in the highest quartile of TyG-BMI, TyG-WC, TyG-WHtR, TyG-BRI, TyG-WWI, and TyG-ABSI demonstrated significantly higher cumulative CVD incidence (all log-rank *p* < 0.001; Figure 2). No significant nonlinear associations were observed for these indices and incident CVD (all *P* for non-linearity > 0.05; Figure 3). Each SD increase in these indices was associated with a 19–32% higher risk of incident CVD, and the HRs comparing the highest with the lowest quartiles ranged from 1.61 to 1.95 (Table 2). Crude CVD incidence rates also increased across quartiles for all TyG-related indices (Table 2). In analyses of individual CVD subtypes, TyG-related indices were generally positively associated with the risks of CHD, stroke, HF, and AF, although the magnitude of association varied across endpoints. The associations were more pronounced for CHD and HF and relatively weaker for stroke and AF (Table 3). These findings suggest that the association with composite incident CVD was not driven by a single CVD subtype. Additional adjustment for baseline hypertension and diabetes did not materially change the associations between TyG-related indices and incident CVD (Supplementary Table S6).

**Table 3:** Associations between TyG-related indices and incident CVD subtypes in participants with depression.

Exposures	CHD	Stroke	HF	AF
	HR (95% CI)	HR (95% CI)	HR (95% CI)	HR (95% CI)
TyG index				
Per SD increment	1.23 (1.20–1.27)	1.04 (0.98–1.10)	1.19 (1.14–1.25)	1.03 (0.99–1.07)
Quartile 1	Reference	Reference	Reference	Reference
Quartile 2	1.25 (1.14–1.38)	1.08 (0.92–1.26)	1.24 (1.06–1.45)	1.02 (0.92–1.14)
Quartile 3	1.47 (1.34–1.61)	1.06 (0.91–1.24)	1.29 (1.10–1.50)	1.05 (0.95–1.17)
Quartile 4	1.76 (1.61–1.92)	1.10 (0.94–1.29)	1.65 (1.43–1.91)	1.04 (0.94–1.16)
TyG-BMI index				
Per SD increment	1.29 (1.26–1.32)	1.07 (1.02–1.13)	1.54 (1.48–1.61)	1.33 (1.29–1.38)
Quartile 1	Reference	Reference	Reference	Reference
Quartile 2	1.31 (1.19–1.44)	1.04 (0.89–1.22)	1.19 (1.00–1.41)	1.02 (0.91–1.15)
Quartile 3	1.56 (1.43–1.71)	1.09 (0.93–1.27)	1.47 (1.25–1.73)	1.22 (1.09–1.36)
Quartile 4	2.00 (1.83–2.18)	1.15 (0.99–1.35)	2.64 (2.27–3.07)	1.74 (1.56–1.93)
TyG-WC index				
Per SD increment	1.35 (1.31–1.39)	1.09 (1.03–1.15)	1.61 (1.54–1.69)	1.36 (1.31–1.41)
Quartile 1	Reference	Reference	Reference	Reference
Quartile 2	1.31 (1.18–1.45)	1.14 (0.97–1.35)	1.25 (1.04–1.50)	1.14 (1.01–1.29)
Quartile 3	1.67 (1.51–1.84)	1.18 (1.00–1.40)	1.79 (1.51–2.13)	1.41 (1.25–1.59)
Quartile 4	2.17 (1.97–2.40)	1.17 (0.99–1.39)	2.90 (2.45–3.43)	1.96 (1.74–2.20)
TyG-WHtR index				
Per SD increment	1.35 (1.31–1.38)	1.10 (1.04–1.16)	1.60 (1.53–1.67)	1.29 (1.24–1.33)
Quartile 1	Reference	Reference	Reference	Reference
Quartile 2	1.33 (1.20–1.48)	1.21 (1.02–1.43)	1.26 (1.05–1.52)	1.00 (0.89–1.13)
Quartile 3	1.67 (1.51–1.84)	1.15 (0.97–1.36)	1.69 (1.42–2.01)	1.24 (1.11–1.39)
Quartile 4	2.21 (2.01–2.43)	1.35 (1.14–1.59)	2.79 (2.37–3.29)	1.61 (1.44–1.80)
TyG-WWI index				
Per SD increment	1.34 (1.30–1.38)	1.11 (1.05–1.17)	1.43 (1.36–1.50)	1.13 (1.08–1.17)
Quartile 1	Reference	Reference	Reference	Reference
Quartile 2	1.48 (1.33–1.64)	1.12 (0.94–1.33)	1.35 (1.13–1.62)	1.13 (1.00–1.27)
Quartile 3	1.73 (1.56–1.91)	1.19 (1.01–1.41)	1.57 (1.32–1.88)	1.18 (1.05–1.32)
Quartile 4	2.26 (2.05–2.49)	1.29 (1.09–1.53)	2.47 (2.09–2.92)	1.35 (1.21–1.51)
TyG-BRI index				
Per SD increment	1.30 (1.27–1.34)	1.10 (1.05–1.16)	1.55 (1.49–1.61)	1.31 (1.27–1.35)
Quartile 1	Reference	Reference	Reference	Reference
Quartile 2	1.37 (1.24–1.51)	1.22 (1.03–1.44)	1.21 (1.01–1.46)	1.05 (0.93–1.18)
Quartile 3	1.69 (1.53–1.86)	1.16 (0.98–1.37)	1.70 (1.43–2.02)	1.28 (1.14–1.43)
Quartile 4	2.22 (2.02–2.44)	1.33 (1.13–1.57)	2.89 (2.45–3.40)	1.77 (1.58–1.98)
TyG-ABSI index				
Per SD increment	1.29 (1.25–1.33)	1.09 (1.03–1.16)	1.29 (1.23–1.36)	1.08 (1.04–1.12)
Quartile 1	Reference	Reference	Reference	Reference
Quartile 2	1.36 (1.23–1.51)	1.19 (1.01–1.41)	1.32 (1.11–1.57)	1.13 (1.01–1.27)
Quartile 3	1.60 (1.44–1.76)	1.15 (0.97–1.37)	1.52 (1.29–1.80)	1.20 (1.07–1.35)
Quartile 4	2.01 (1.81–2.22)	1.25 (1.05–1.49)	2.00 (1.69–2.37)	1.24 (1.10–1.40)

Models were adjusted for age, sex, race, employment status, educational level, Townsend deprivation index, smoking status, drinking frequency, sleep duration, and physical activity. CHD, coronary heart disease; AF, atrial fibrillation; HF, heart failure; SD, standard deviation; HR, hazard ratio; CI, confidence interval; TyG, triglyceride-glucose; BMI, body mass index; WC, waist circumference; WHtR, waist-to-height ratio; BRI, body roundness index; ABSI, a body shape index; WWI, weight-adjusted waist index.

### 3.3 Associations of TyG-related indices with mortality

Over a median follow-up of 13.7 years (IQR: 13.0–14.5), 546 CVD deaths and 4,619 all-cause deaths occurred. KM curves indicated significantly higher cumulative mortality among participants in the highest quartile of the TyG index (all log-rank *p* < 0.001; Supplementary Figures S2B,C). RCS analyses showed no evidence of nonlinearity for CVD mortality (*P* for non-linearity = 0.790; Supplementary Figure S2E), whereas a significant U-shaped relationship was observed for all-cause mortality (*P* for non-linearity < 0.001; Supplementary Figure S2F). Each SD increase in the TyG index was associated with higher risks of CVD mortality (HR = 1.18, 95% CI: 1.09–1.29) and all-cause mortality (HR = 1.03, 95% CI: 1.01–1.06) (Table 2). Based on the observed dose–response patterns, the first and second quartiles (Q1 and Q2) of the TyG index were selected as reference groups for CVD mortality and all-cause mortality, respectively. Compared with the respective reference group, participants in the highest quartile had elevated risks of CVD mortality (HR: 1.64; 95% CI: 1.24–2.16) and all-cause mortality (HR: 1.12; 95% CI: 1.03–1.21) (Table 2). The crude CVD mortality rate increased from 0.4 to 1.1 deaths per 1,000 person-years across TyG quartiles, while the crude all-cause mortality rate increased from 4.9 to 8.2 deaths per 1,000 person-years (Table 2).

Consistent mortality associations were observed for the other TyG-related indices. Participants in the highest quartiles exhibited significantly higher cumulative risks of mortality (all log-rank *p* < 0.001; Supplementary Figures S3, S4). For CVD mortality, RCS analyses showed no significant departure from linearity for any TyG-related index (all *P* for non-linearity > 0.05; Supplementary Figures S5A–F). Each SD increment in TyG-BMI, TyG-WC, TyG-WHtR, TyG-WWI, TyG-BRI, and TyG-ABSI was associated with a 24–37% higher risk of CVD mortality, and the HRs comparing the highest with the lowest quartiles ranged from 1.59 to 2.30 (Table 2). The crude CVD mortality rates increased from 0.3–0.5 deaths per 1,000 person-years in the lowest quartiles to 1.1–1.5 deaths per 1,000 person-years in the highest quartiles of these indices (Table 2). For all-cause mortality, nonlinear associations were observed for most TyG-related indices (all *P* for non-linearity < 0.05; Supplementary Figures S5G–K), except for TyG-ABSI (*P* for nonlinearity = 0.608; Supplementary Figure S5L). Each SD increment in these indices was associated with a 7–16% higher risk of all-cause mortality (Table 2). Compared with the Q2 reference group, the HRs for all-cause mortality in Q4 ranged from 1.23 to 1.37 for TyG-BMI, TyG-WC, TyG-WHtR, TyG-WWI, and TyG-BRI. For TyG-ABSI, compared with Q1, the HRs increased across Q2–Q4 from 1.14 to 1.43 (Table 2). The crude all-cause mortality rates in the Q2 reference groups were 5.4–5.9 deaths per 1,000 person-years, whereas the corresponding rates in Q4 were 8.0–10.1 deaths per 1,000 person-years. Associations between TyG-related indices and mortality remained materially unchanged after additional adjustment for baseline hypertension and diabetes (Supplementary Table S6). Crude association estimates are provided in Supplementary Table S7. Compared with crude models, multivariable-adjusted estimates were generally attenuated but remained directionally consistent.

### 3.4 Incremental predictive information provided by TyG-related indices

Table 4 summarizes changes in model performance after adding each TyG-related index to the conventional cardiovascular risk model. Overall, except for the TyG index and TyG-BMI, the addition of other TyG-related indices showed statistically significant but modest improvements in discrimination and risk reclassification. However, the absolute magnitude of improvement was small, and the relative performance of individual indices varied across the NRI, IDI, and C-index. These findings should be interpreted as modest incremental predictive information rather than evidence of clinically meaningful improvement, demonstrated clinical utility, or superiority of any single index.

**Table 4:** Increment predictive values of TyG-related indices for incident CVD and mortality in participants with depression.

Models	NRI, %		IDI, %		C-index	
	Estimate (95% CI)	*P*-value	Estimate (95% CI)	*P*-value	Estimate (95% CI)	*P*-value
Incident CVD						
Conventional model	Reference		Reference		0.7041 (0.6990, 0.7093)	–
Conventional model + TyG index	1.402 (−2.100, 2.657)	0.248	0.001 (−0.002, 0.013)	0.940	0.7041 (0.6990, 0.7093)	0.321
Conventional model + TyG-BMI index	0.614 (−1.283, 1.848)	0.442	0.001 (−0.001, 0.013)	0.968	0.7041 (0.6990, 0.7093)	0.277
Conventional model + TyG-WC index	4.014 (2.788, 5.300)	<0.001	0.077 (0.032, 0.140)	0.005	0.7049 (0.6997, 0.7100)	0.001
Conventional model + TyG-WHtR index	3.877 (2.711, 5.170)	<0.001	0.054 (0.017, 0.115)	0.031	0.7048 (0.6997, 0.7099)	<0.001
Conventional model + TyG-WWI index	4.306 (3.163, 5.565)	<0.001	0.062 (0.025, 0.122)	0.013	0.7049 (0.6998, 0.7100)	<0.001
Conventional model + TyG-ABSI index	4.604 (3.382, 5.877)	<0.001	0.084 (0.036, 0.149)	0.004	0.7050 (0.6999, 0.7101)	<0.001
Conventional model + TyG-BRI index	2.171 (0.819, 3.349)	<0.001	0.099 (0.048, 0.168)	0.001	0.7052 (0.7000, 0.7103)	<0.001
All-cause mortality						
Conventional model	Reference		Reference		0.7252 (0.7183, 0.7322)	–
Conventional model + TyG index	3.065 (0.537, 4.698)	0.004	0.009 (−0.003, 0.042)	0.443	0.7255 (0.7186, 0.7325)	0.066
Conventional model + TyG-BMI index	3.461 (1.610, 4.844)	<0.001	0.015 (−0.002, 0.056)	0.308	0.7256 (0.7186, 0.7325)	0.057
Conventional model + TyG-WC index	7.020 (5.223, 8.353)	<0.001	0.202 (0.114, 0.321)	<0.001	0.7273 (0.7204, 0.7343)	<0.001
Conventional model + TyG-WHtR index	7.447 (5.934, 9.011)	<0.001	0.298 (0.183, 0.434)	<0.001	0.7283 (0.7214, 0.7352)	<0.001
Conventional model + TyG-WWI index	7.823 (6.154, 9.352)	<0.001	0.324 (0.202, 0.459)	<0.001	0.7287 (0.7217, 0.7356)	<0.001
Conventional model + TyG-ABSI index	7.848 (6.349, 9.540)	<0.001	0.316 (0.205, 0.464)	<0.001	0.7287 (0.7218, 0.7356)	<0.001
Conventional model + TyG-BRI index	7.024 (5.322, 8.505)	<0.001	0.504 (0.348, 0.680)	<0.001	0.7300 (0.7231, 0.7369)	<0.001
CVD mortality						
Conventional model	Reference		Reference		0.7830 (0.7659, 0.8002)	–
Conventional model + TyG index	3.707 (−4.949, 7.969)	0.401	0.003 (−0.009, 0.060)	0.611	0.7834 (0.7663, 0.8005)	0.333
Conventional model + TyG-BMI index	3.715 (−4.218, 8.825)	0.359	0.002 (−0.009, 0.071)	0.676	0.7834 (0.7663, 0.8005)	0.308
Conventional model + TyG-WC index	7.279 (1.658, 11.616)	0.011	0.059 (0.004, 0.216)	0.034	0.7853 (0.7683, 0.8023)	0.120
Conventional model + TyG-WHtR index	8.844 (3.904, 13.201)	<0.001	0.105 (0.022, 0.316)	0.013	0.7867 (0.7698, 0.8036)	0.084
Conventional model + TyG-WWI index	9.365 (4.718, 13.580)	<0.001	0.099 (0.014, 0.280)	0.022	0.7869 (0.7700, 0.8037)	0.080
Conventional model + TyG-ABSI index	8.282 (3.823, 12.949)	<0.001	0.084 (0.011, 0.252)	0.025	0.7865 (0.7696, 0.8034)	0.078
Conventional model + TyG-BRI index	7.147 (2.276, 11.447)	0.004	0.256 (0.085, 0.654)	0.003	0.7890 (0.7723, 0.8058)	0.021

Conventional models were adjusted for age, sex, smoking status, systolic blood pressure, hypertension medication, diabetes, total cholesterol, and high-density lipoprotein cholesterol. CI, confidence interval; CVD, cardiovascular disease; TyG, triglyceride-glucose; BMI, body mass index; WC, waist circumference; WHtR, waist-to-height ratio; BRI, body roundness index; ABSI, a body shape index; WWI, weight-adjusted waist index; NRI, net reclassification index; IDI, integrated discrimination improvement index.

For incident CVD, adding TyG-BRI, TyG-ABSI, and TyG-WWI improved all evaluated metrics, with continuous NRI ranging from 2.17 to 4.60% (all *p* < 0.001) and IDI ranging from 0.054 to 0.099% (all *p* < 0.05). These indices also produced statistically significant but very small increases in the C-index (all *p* ≤ 0.001). For example, the C-index increased from 0.7041 in the conventional model to 0.7052 after adding TyG-BRI. Calibration analyses at the 10-year time horizon showed generally good agreement between predicted and observed incident CVD risks, but the calibration curves for the conventional and expanded models were largely overlapping, indicating that adding TyG-related indices did not materially alter model calibration (Supplementary Figure S6). For all-cause and CVD mortality, adding several TyG-related indices also resulted in statistically significant but modest improvements in prediction metrics. Nevertheless, the relative ranking of individual indices differed across NRI, IDI, and C-index. Taken together, these findings suggest that several TyG-related indices provide limited additional predictive information beyond conventional cardiovascular risk factors. Given the small absolute increases in discrimination, largely overlapping calibration curves, and metric-dependent differences, these results should not be interpreted as demonstrating clinical utility or supporting clinical implementation. External validation and implementation-focused studies are needed before TyG-related indices can be considered for use in cardiovascular risk prediction among individuals with depression.

### 3.5 Exploratory biomarker-based analyses for incident CVD

**Figure 4:**
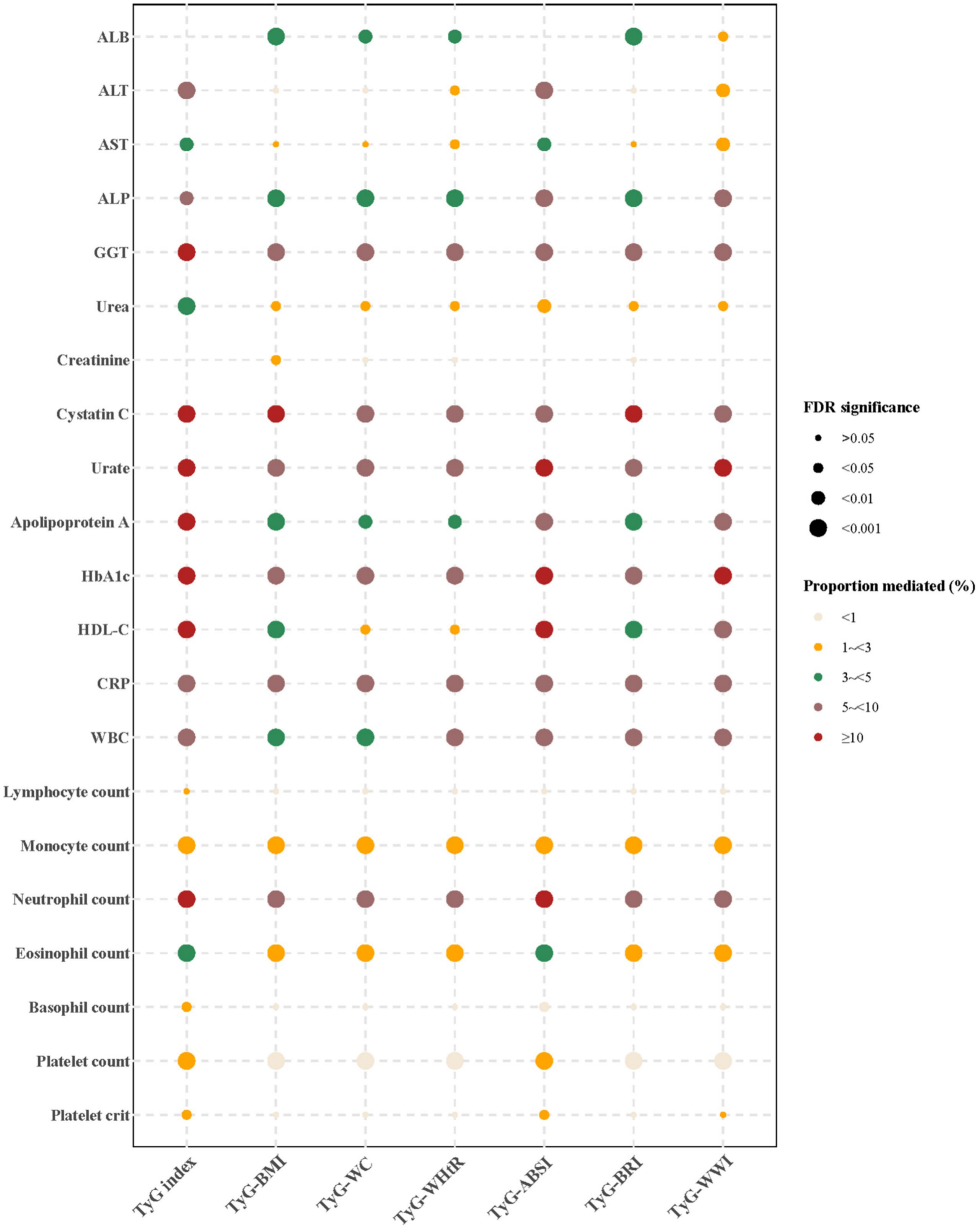
Proportion mediated by selected biomarkers as potential mediators in the associations between TyG-related indices and incident CVD in participants with depression. Models were adjusted for age, sex, race, employment status, educational level, Townsend deprivation index, smoking status, drinking frequency, sleep duration, and physical activity. FDR, false discovery ratio; ALB, albumin; ALT, alanine aminotransferase; AST, aspartate aminotransferase; ALP, alkaline phosphatase; GGT, gamma glutamyl transferase; CRP, C-reactive protein; HbA1c, glycated hemoglobin; WBC, white blood cell count; TyG, triglyceride-glucose; BMI, body mass index; WC, waist circumference; WHtR, waist-to-height ratio; BRI, body roundness index; ABSI, a body shape index; WWI, weight-adjusted waist index.

We examined the associations of TyG-related indices with 29 baseline biomarkers related to liver function, renal function, metabolic regulation, and systemic inflammation, and further explored whether these biomarkers statistically accounted for part of the associations between TyG-related indices and incident CVD. Biomarkers showing significant and directionally consistent associations in both linear regression and Cox models were included in the exploratory biomarker analyses. After FDR correction, 19–21 biomarkers were evaluated across the TyG-related indices (all FDR-adjusted *p* < 0.05; Supplementary Tables S8, S9). Figure 4 and Supplementary Table S10 summarize the estimated proportions statistically accounted for by selected candidate biomarkers.

Among liver function biomarkers, ALP [4.6% (95% CI: 3.5–5.9) to 9.1% (95% CI: 7.0–12.0)] and GGT [5.0% (95% CI: 3.6–6.4) to 12.3% (95% CI: 9.4–15.9)] showed the most consistent contributions (all FDR-adjusted *p* < 0.001). In contrast, ALT and AST showed relatively smaller statistical contributions (generally <5%). Among renal function biomarkers, urate and cystatin C showed notable contributions, with estimates ranging from 8.6 to 25.8% and from 9.5 to 11.7%, respectively. Among metabolic biomarkers, HbA1c and HDL-C also statistically accounted for part of the observed associations, with estimates ranging from 6.5 to 23.9% and from 4.9 to 27.4%, respectively. Among inflammatory biomarkers, CRP, WBC, and neutrophils also showed statistically significant contributions across all indices, with estimates ranging from 7.5 to 9.2%, from 4.6 to 8.9%, and from 6.5 to 13.1%, respectively. Overall, these exploratory biomarker analyses suggest that baseline biomarkers reflecting hepatic and renal function, metabolic regulation, and systemic inflammation may represent possible biological correlates or candidate intermediary biomarkers related to TyG-associated cardiovascular risk. However, these findings should not be interpreted as causal mediation or confirmed mechanistic pathways.

### 3.6 Sensitivity and subgroup analyses

Sensitivity and subgroup analyses supported the robustness of the observed associations. The associations between TyG-related indices and study outcomes remained materially unchanged after excluding events that occurred within the first 2 years of follow-up (Supplementary Table S11) and after applying multiple imputation for covariates with missing values (Supplementary Table S12). Results were also broadly consistent when stricter depression definitions were applied, including ICD-10-coded depression and PHQ-2-defined probable depression (Supplementary Table S13). In competing-risk analyses, the associations between TyG-related indices and incident CVD and CVD mortality were materially consistent with those from the primary Cox models after treating non-CVD death as a competing event (Supplementary Table S14). Subgroup analyses stratified by age, sex, race, educational attainment, employment status, TDI, smoking status, drinking frequency, PA, and sleep duration also yielded broadly consistent results (Supplementary Table S15).

## 4 Discussion

In this large prospective cohort of 53,171 individuals with depression followed for more than 13 years, higher levels of all seven TyG-related indices were consistently associated with elevated risks of incident CVD, CVD mortality, and all-cause mortality. Each SD increase in TyG-related indices was associated with a 19–32% higher risk of incident CVD, a 24–37% higher risk of CVD mortality, and a 7–16% higher risk of all-cause mortality. These associations were generally robust across multiple sensitivity and subgroup analyses. No significant departure from linearity was observed for the associations with incident CVD and CVD mortality. When added to a conventional risk model, several TyG-related indices provided statistically significant but modest incremental predictive information. Exploratory biomarker analyses further identified biomarkers related to metabolic dysregulation, hepatic and renal function, and systemic inflammation as possible biological correlates of the observed associations.

Previous studies have established the prognostic value of TyG-related indices in several high-risk somatic populations (27–32). However, evidence in mental health populations remains limited, although depression is a recognized independent risk factor for CVD incidence and mortality (7–10). To date, only two prior studies have examined TyG-related indices among individuals with depression, both focusing on all-cause mortality and using smaller sample sizes (36, 37). In contrast, the present study comprehensively evaluated seven TyG-related indices in relation to incident CVD, CVD mortality, and all-cause mortality in a large cohort of individuals with depression. In line with earlier investigations (27–32, 36, 37), all TyG-related indices were positively associated with adverse cardiovascular and mortality outcomes. Consistent with prior observations (29, 56), our findings demonstrated positive associations between TyG-related indices and CVD outcomes, with no significant departure from linearity. In contrast, the nonlinear dose–response associations observed between most TyG-related indices and all-cause mortality are consistent with a previous long-term cohort study of individuals with depression (37), whereas another study reported a linear association, possibly due to its shorter follow-up duration of only 5 years (36). The strength of associations differed across CVD subtypes, with relatively stronger associations observed for CHD and HF and weaker associations for stroke and AF. These differences may reflect variation in underlying pathophysiology. TyG-related indices reflect IR-related metabolic dysfunction and, for anthropometric derivatives, combined metabolic-adiposity burden. IR may promote atherosclerotic CVD through dyslipidemia, endothelial dysfunction, oxidative stress, inflammation, and vascular remodeling (57–59), which are central to the development and progression of CHD (58, 59). IR and excess adiposity may also contribute to HF through altered myocardial insulin signaling, impaired substrate metabolism, mitochondrial dysfunction, myocardial lipotoxicity, microvascular dysfunction, neurohormonal activation, and adverse cardiac remodeling (60, 61). By contrast, the weaker associations observed for stroke and AF may reflect the greater etiological heterogeneity of these outcomes. Stroke includes ischemic and hemorrhagic subtypes, and ischemic stroke itself may arise from large-artery atherosclerosis, small-vessel disease, and other mechanisms (62, 63). Similarly, AF is influenced by multiple factors beyond metabolic dysfunction, including age, hypertension, structural heart disease, valvular disease, obesity, obstructive sleep apnea, alcohol intake, and genetic susceptibility (64–66). Thus, TyG-related indices may capture only part of the risk profile for these more heterogeneous cardiovascular outcomes. Overall, our findings suggest that greater metabolic-adiposity burden, as reflected by TyG-related indices, is associated with higher cardiovascular risk in individuals with depression.

Beyond association analyses, we examined whether TyG-related indices provided incremental predictive information when added to conventional cardiovascular risk factors. Consistent with prior studies suggesting that combining TyG with obesity-related indicators may provide additional cardiovascular risk information (29–32, 56), we observed statistically significant improvements in NRI, IDI, and C-index. However, because the conventional model already included diabetes and lipid-related variables, the modest incremental value of TyG-related indices may partly reflect a compact representation of existing metabolic-adiposity information rather than a substantially novel risk signal. Recent studies have evaluated TyG combined with novel adiposity-related indicators such as WWI, ABSI, and BRI (32, 56, 67, 68). Some studies reported that TyG-WWI or TyG-ABSI showed relatively favorable performance for cardiovascular risk assessment (32, 67, 68). In the present study, several novel TyG-related indices showed relatively larger numerical increments in some prediction metrics than traditional indices, but their relative performance varied across outcomes and metrics. For example, TyG-BRI showed relatively larger numerical increments in IDI and C-index for some outcomes, whereas TyG-ABSI showed relatively larger NRI for incident CVD and all-cause mortality. These findings do not support a single best-performing index but suggest that TyG-related composite indices may reflect combined metabolic and adiposity-related burden relevant to cardiovascular risk in individuals with depression. Furthermore, because depression is clinically heterogeneous, future cardiovascular risk models may benefit from integrating biological markers with symptom-based profiles, including clinically meaningful dimensions such as anhedonia (69). The low cost and availability of TyG-related indices may make them attractive candidate markers for cardiovascular risk assessment in psychiatric populations, particularly within integrated care settings that emphasize metabolic monitoring and lifestyle-oriented management (70). Nevertheless, the observed gains in discrimination were small, calibration curves for the conventional and expanded models were largely overlapping. Therefore, their clinical utility remains to be established through external validation, decision-curve analysis, and implementation-focused studies before routine clinical use can be considered.

To explore possible biological correlates, we conducted exploratory mediation analyses using baseline biomarkers. Liver-related biomarkers, particularly ALP and GGT, statistically accounted for part of the observed associations. Previous studies have documented associations between TyG-related indices and liver enzymes (71, 72), and elevated liver function biomarkers have also been associated with higher CVD risk (73). These findings may reflect the coexistence of hepatic metabolic dysfunction, lipid accumulation, inflammatory activation, and adverse cardiometabolic status (74). Renal biomarkers, particularly urate and cystatin C, also showed statistical contributions. Prior observational studies have linked TyG-related indices to impaired renal function (75), while reduced kidney function has been associated with cardiovascular events (76, 77). IR may be associated with renal injury through endothelial dysfunction, oxidative stress, inflammatory activation, and coagulation abnormalities (78, 79). Conversely, impaired renal function may coexist with systemic inflammation, metabolic imbalance, and arterial stiffness, which are all associated with cardiovascular risk (80). Inflammatory markers, including CRP, WBC, and neutrophil count, showed statistical significance but modest contributions. IR has been associated with pro-inflammatory signaling, increased cytokine production, chronic low-grade inflammation, and oxidative stress (57, 81), which may coexist with endothelial dysfunction, myocardial ischemia, and atherosclerotic progression, and are consistent with proposed pathways linking metabolic dysfunction to CVD (57, 81). Likewise, prior studies have reported that inflammatory markers may statistically account for part of the associations between TyG-related indices and cardiovascular outcomes (27, 30, 82). Metabolic biomarkers, including HbA1c and HDL-C, highlighted the relevance of glycemic regulation and lipid metabolism in these associations (26). Depression and IR may also share biological processes, including HPA axis dysregulation, immune activation, and gut microbiota-related metabolic and neurotransmitter pathways (22, 23). Overall, these biomarker patterns suggest that hepatic and renal function, systemic inflammation, and metabolic regulation may be biological correlates of TyG-related cardiovascular risk among individuals with depression.

The current study provides novel prospective evidence linking TyG-related indices to incident CVD, CVD mortality, and all-cause mortality in individuals with depression. Notable strengths include the large sample size, extended follow-up, rigorous covariate adjustment, comprehensive sensitivity and subgroup analyses, and the availability of detailed biomarker data for exploratory analyses of candidate biological correlates. Several limitations warrant consideration. First, despite the established validity of association analyses (83), the “healthy volunteer” bias of the UK Biobank may limit generalizability (84), and the predominantly White study population may further restrict applicability to other ethnic groups. Second, excluding participants with missing TyG-related indices may have introduced selection bias, although baseline comparisons showed limited imbalance between included and excluded participants, with most standardized mean differences being small (Supplementary Table S16). Third, the observational design precludes causal inference, and reverse causation and residual confounding cannot be excluded. The U-shaped associations observed for all-cause mortality may partly reflect reverse causation, as individuals in the lowest quartile may include those with undiagnosed wasting diseases, frailty, or other preclinical conditions associated with increased mortality. Residual confounding by depression-related clinical factors and psychotropic treatment burden is also possible because detailed information on depression severity, chronicity, recurrence, symptom profile, psychotherapy, antipsychotic exposure, mood stabilizer use, and cumulative psychotropic medication burden was unavailable. Antidepressant use may have influenced both TyG-related metabolic profiles and cardiovascular outcomes through effects on body weight, adiposity, glucose metabolism, lipid profiles, blood pressure, and other cardiometabolic risk factors (15, 16). It may also serve as a marker of more severe, recurrent, or chronic depression, leading to potential confounding by indication. Fourth, TyG-related indices were measured only at baseline, which may lead to exposure misclassification and regression dilution bias during the long follow-up. Fifth, the incremental prediction analyses were conducted within the same cohort without formal external validation. Thus, improvements in C-index, NRI, and IDI may be optimistic, and numerical differences among indices should be interpreted as descriptive rather than definitive comparative evidence. Sixth, the integrated depression definition may have introduced clinical heterogeneity and potential misclassification. Although this approach improved case ascertainment by combining symptom-based, self-reported, medication-based, and hospital-record-based evidence of depression, these sources may capture different clinical phenotypes. Such heterogeneity may mix individuals with different levels of depression severity, chronicity, recurrence, treatment exposure, comorbidity burden, and healthcare engagement, which could influence both TyG-related indices and cardiovascular outcomes. Although sensitivity analyses using stricter depression definitions yielded broadly consistent results, they could not fully eliminate potential bias related to heterogeneous depression ascertainment. Finally, TyG-related indices and candidate intermediary biomarkers were assessed at the same baseline visit, preventing the determination of temporal ordering. Future studies using structured diagnostic interviews, detailed longitudinal treatment data, and repeated biomarker measurements, and external validation are needed to confirm these findings and clarify their clinical and biological implications.

## 5 Conclusion

In this large prospective cohort study of individuals with depression, higher levels of seven TyG-related indices were consistently associated with increased risks of incident CVD, CVD mortality, and all-cause mortality. Several TyG-related indices provided statistically significant but modest incremental predictive information beyond conventional cardiovascular risk factors, support their potential role as candidate metabolic-adiposity markers for characterizing cardiovascular risk profiles in individuals with depression. Exploratory biomarker analyses identified inflammatory, metabolic, hepatic, and renal biomarkers as possible biological correlates of the observed associations.

## Data Availability

The datasets supporting this study were sourced from UK Biobank (https://www.ukbiobank.ac.uk). Access is restricted due to licensing agreements. Data requests can be obtained from the authors upon reasonable request, and with the approval of UK Biobank. Requests to access the datasets should be directed to the corresponding author.
