# Supplementary material for: Triglyceride-glucose-related indices and cardiovascular disease and mortality among individuals with depression: a prospective study from UK Biobank
Source: Front Nutr. 2026 Jun 23;13:1842720. doi: 10.3389/fnut.2026.1842720 (PMC13337923; doi:10.3389/fnut.2026.1842720)
Supplement: Supplementary file 1 [file Supplementary_file_1.DOCX]

**Triglyceride-glucose-related indices and cardiovascular disease and mortality among individuals with depression: a prospective study from UK Biobank**

**Supplementary materials**

[**Table S1. Definition of participants with depression at baseline identified from multiple data sources 3**](#_Toc230642387)

[**Table S2. Detailed information of blood biomarkers in UK Biobank 5**](#_Toc230642388)

[**Table S3. Summary of missing information on covariates 7**](#_Toc230642389)

[**Table S4. Measures of covariates at baseline in the UK Biobank. 8**](#_Toc230642390)

[**Table S5. Baseline characteristics of study population stratified by the incidence of overall CVD 11**](#_Toc230642391)

[**Table S6. Sensitivity analysis for the associations between TyG-related indices and incident CVD and mortality in participants with depression: additionally adjusting for hypertension and diabetes. 13**](#_Toc230642392)

[**Table S7. Crude associations of TyG-related indices with incident CVD, CVD mortality, and all-cause mortality. 16**](#_Toc230642393)

[**Table S8. Associations between TyG-related indices and selected biomarkers in individuals with depression 18**](#_Toc230642394)

[**Table S9. Associations between selected biomarkers and risk of cardiovascular disease in individuals with depression 27**](#_Toc230642395)

[**Table S10. Proportion mediated by selected biomarkers as potential mediators in the associations between TyG-related indices and incident CVD in participants with depression 29**](#_Toc230642396)

[**Table S11. Sensitivity analysis for the associations between TyG-related indices and incident CVD and mortality in participants with depression: excluding participants who developed outcomes within the first 2 years of follow-up 31**](#_Toc230642397)

[**Table S12. Sensitivity analysis for the associations between TyG-related indices and incident CVD and mortality in participants with depression: imputing covariates with missing values using multiple imputation approach 34**](#_Toc230642398)

[**Table S13. Sensitivity analysis for the associations between TyG-related indices and incident CVD in participants with depression: using stricter definitions of baseline depression 37**](#_Toc230642399)

[**Table S14. Sensitivity analysis for the associations between TyG-related indices and incident CVD and mortality in participants with depression: using the Fine–Gray competing risk model. 39**](#_Toc230642400)

[**Table S15. Subgroup analysis for association between TyG-related indices and incident CVD in individuals with depression 41**](#_Toc230642401)

[**Table S16. Baseline characteristics of participants with complete TyG-related indices and those excluded because of missing TyG-related indices among participants with depression and without baseline CVD. 47**](#_Toc230642402)

[**Figure S1. Directed acyclic graph illustrating the adjusted framework for the association between TyG-related indices and cardiovascular outcomes. 49**](#_Toc230642403)

[**Figure S2. Kaplan-Meier curves of study outcomes according to TyG index quartiles (A-C), and dose-response relationship of the TyG index with outcomes by RCS analysis in participants with depression (D-F). 50**](#_Toc230642404)

[**Figure S3. Kaplan-Meier curves of CVD mortality according to the quartiles of TyG-related indices in participants with depression. 51**](#_Toc230642405)

[**Figure S4. Kaplan-Meier curves of all-cause mortality according to the quartiles of TyG-related indices in participants with depression. 52**](#_Toc230642406)

[**Figure S5. Dose-response relationship of TyG-related indices with CVD and all-cause mortality by RCS analysis in participants with depression. 53**](#_Toc230642407)

[**Figure S6. Calibration plots for 10-year incident CVD risk 54**](#_Toc230642408)

#

# Table S1. Definition of participants with depression at baseline identified from multiple data sources

| **Classification** | **Source** | **Criteria** | **Field ID or ICD-10 code** |
| --- | --- | --- | --- |
| Self-reported depressive symptoms | Patient Health Questionnaire-2 (PHQ-2) | Participants were classified as having depression if PHQ-2 score ≥ 3 | 2050 and 2060 |
| Medication use | Treatment/medication code | Self-reported antidepressant medication use at baseline | 20003 (1140879616, 1140921600, 1140879540, 1140867878, 1140916282, 1140909806, 1140867888, 1141152732, 1141180212, 1201, 1140879634, 1140867876, 1140882236, 1141200564, 1141190158, 1140867726, 1140879620, 1140867818, 1140879630, 1140916288, 1140879628, 1141151946, 1140867624, 1140867756, 1140867884, 1141151978, 1141152736, 1141201834, 1140867690, 1140867640, 1140867920, 1141200570, 1140867850, 1140879544, 1141174756, 1140867758, 1140867960, 1141176858, 1140867914, 1141176854, 1140867820) |
| Self-reported diagnosis at baseline | Non-cancer illness code, self-reported | Self-reported depression diagnosis at baseline | 20002 (1286, 1531) |
| Clinical diagnosis | A clinical diagnosis of depression at hospital admission records before recruitment. | Diagnosed depressive disorders based on ICD-10 codes, obtained from: Health Episode Statistics (England & Wales) and Scottish Morbidity Records (Scotland) | F32 and F33 |

**Table S2. Detailed information of blood biomarkers in UK Biobank**

| **Field ID** | **Blood biomarkers** | **Unit** | **Classification** |
| --- | --- | --- | --- |
| 30600 | Albumin (ALB) | U/L | Liver function |
| 30620 | Alanine aminotransferase (ALT) | U/L | Liver function |
| 30650 | Aspartate aminotransferase (AST) | umol/L | Liver function |
| 30660 | Direct bilirubin (DB) | U/L | Liver function |
| 30730 | Gamma glutamyltransferase (GGT) | umol/L | Liver function |
| 30840 | Total bilirubin | g/L | Liver function |
| 30860 | Total protein | g/L | Liver function |
| 30610 | Alkaline phosphatase (ALP) | U/L | Liver function |
| 30670 | Urea | mmol/L | Renal function |
| 30700 | Creatinine | umol/L | Renal function |
| 30720 | Cystatin C | mg/L | Renal function |
| 30810 | Phosphate | mmol/L | Renal function |
| 30880 | Urate | umol/L | Renal function |
| 30630 | Apolipoprotein A | g/L | Metabolic |
| 30640 | Apolipoprotein B | g/L | Metabolic |
| 30690 | Cholesterol | mmol/L | Metabolic |
| 30750 | Glycated hemoglobin (HbA1c) | mmol/mol | Metabolic |
| 30760 | HDL cholesterol (HDL-C) | mmol/L | Metabolic |
| 30780 | LDL direct (LDL-C) | mmol/L | Metabolic |
| 30790 | Lipoprotein A | nmol/L | Metabolic |
| 30710 | C-reactive protein (CRP) | mg/L | Inflammatory |
| 30000 | White blood cell count (WBC) | 10^9 cells/Litre | Inflammatory |
| 30120 | Lymphocyte count | 10^9 cells/Litre | Inflammatory |
| 30130 | Monocyte count | 10^9 cells/Litre. | Inflammatory |
| 30140 | Neutrophil count | 10^9 cells/Litre | Inflammatory |
| 30150 | Eosinophil count | 10^9 cells/Litre | Inflammatory |
| 30160 | Basophil count | 10^9 cells/Litre | Inflammatory |
| 30080 | Platelet count | 10^9 cells/Litre | Inflammatory |
| 30090 | Platelet crit | 10^9 cells/Litre | Inflammatory |

**Table S3. Summary of missing information on covariates**

| **Covariates** | **Number (%)** |
| --- | --- |
| Age | 0 (0) |
| Sex | 0 (0) |
| Ethnicity | 247 (0.44) |
| Employment | 313 (0.55) |
| Educational level | 710 (1.25) |
| Townsend deprivation index | 95 (0.17) |
| Physical activity | 1,872 (3.30) |
| Smoking | 264 (0.47) |
| Drinking | 141 (0.25) |
| Sleep duration | 734 (1.29) |

**Table S4. Measures of covariates at baseline in the UK Biobank.**

| **Variable** | **Filed ID** | **Question or description** | **Categories from raw data** | **Categories for the current study** |
| --- | --- | --- | --- | --- |
| Sex | 31 | Biological sex | Female  Male | 0 = Female  1= Male |
| Age | 21022 | Age at recruitment | Continuous. |  |
| Ethnicity | 21000 | Ethnic background | White;  Mixed;  Asian or Asian British;  Black or Black British;  Chinese;  Other ethnic group. | 0 = White;  1 = Non-White |
| Education | 6138 | Qualifications | College or University degree;  A levels/AS levels or equivalent;  O levels/GCSEs or equivalent;  CSEs or equivalent;  NVQ or HND or HNC or equivalent;  Other professional qualifications eg: nursing, teaching. | 0 = College or university degree;  1 = Others. |
| Employment | 6142 | Current employment status | In paid employment or self-employed;  Retired;  Looking after home and/or family;  Unable to work because of sickness or disability;  Unemployed;  Doing unpaid or voluntary work;  Full or part-time student;  None of the above. | 0 = Employed (including those paid employment or self-employed, retired, doing unpaid or voluntary work, or being full or part time students);  1 = Unemployed. |
| Townsend deprivation index | 22189 | Townsend deprivation index at recruitment | Continuous. | A higher value indicates greater deprivation. |
| Smoking | 20116 | The current/past smoking status of the participant. | Never; Past; Current. | 0 = Never smoker;  1 = Ever smoker;  2= Current smoker. |
| Alcohol consumption | 1558 | About how often do you drink alcohol? | Never; Special occasions only; One to three times a month; Once or twice a week; Three or four times a week; Daily or almost daily. | 0 = Never drinking;  1 = Special occasions only;  2 = One to three time a month;  3 = Oner or twice a week;  4 = Three or four times a week;  5 = Daily or almost daily. |
| Adequate physical activity | 884,894,904,914 | Number of days/week of moderate/vigorous physical activity 10+ minutes?  Duration of moderate/vigorous activity? | Continuous. | Total amount of weekly moderate and vigorous physical activity was calculated by multiplying the number of days per week and the duration per day, respectively. Adequate physical activity was defined as vigorous physical activity ≥75 min/week, or moderate physical activity ≥150 min/week, or equivalent combination.  0 = Adequate; 1 = Unadequate. |
| Sleep duration | 1160 | About how many hours sleep do you get in every 24 hours? (please include naps)? | Continuous. | 0 = 7-8 hours/day;  1 = <7 hours/day or >8 hours/day. |

**Table S5.** **Baseline characteristics of study population stratified by the incidence of overall CVD**

|  | **Total population** | **CVD incidence** | | ***P* values** |
| --- | --- | --- | --- | --- |
|  |  | **No** | **Yes** |  |
| *n* | 53,171 | 44,655 | 8,516 |  |
| Age (years) | 56.00 (49.00, 62.00) | 55.00 (48.00, 61.00) | 60.00 (54.00, 65.00) | <0.001 |
| Sex |  |  |  | <0.001 |
| Male | 18,232 (34.3) | 14,316 (32.1) | 3,916 (46.0) |  |
| Female | 34,939 (65.7) | 30,339 (67.9) | 4,600 (54.0) |  |
| Ethnicity |  |  |  | <0.001 |
| White | 49,940 (93.9) | 41,854 (93.7) | 8,086 (95.0) |  |
| Non-white | 3,231 (6.1) | 2,801 (6.3) | 430 (5.0) |  |
| Employed status |  |  |  | <0.001 |
| Employed | 44,532 (83.8) | 37,589 (84.2) | 6,943 (81.5) |  |
| Not employed | 8,639 (16.2) | 7,066 (15.8) | 1,573 (18.5) |  |
| Educational level |  |  |  | <0.001 |
| University or college | 15,988 (30.1) | 13,928 (31.2) | 2,060 (24.2) |  |
| Others | 37,183 (69.9) | 30,727 (68.8) | 6,456 (75.8) |  |
| Townsend deprivation index | -1.69 (-3.41, 1.36) | -1.76 (-3.45, 1.26) | -1.32 (-3.21, 1.95) | <0.001 |
| Physical activity |  |  |  | <0.001 |
| Adequate | 29,841 (56.1) | 25,270 (56.6) | 4,571 (53.7) |  |
| Inadequate | 23,330 (43.9) | 19,385 (43.4) | 3,945 (46.3) |  |
| Smoking status |  |  |  | <0.001 |
| Never smoking | 27,191 (51.1) | 23,453 (52.5) | 3,738 (43.9) |  |
| Ever smoking | 17,842 (33.6) | 14,691 (32.9) | 3,151 (37.0) |  |
| Current smoking | 8,138 (15.3) | 6,511 (14.6) | 1,627 (19.1) |  |
| Drinking frequency |  |  |  | <0.001 |
| Never | 6,317 (11.9) | 5,088 (11.4) | 1,229 (14.4) |  |
| Special occasions only | 8,076 (15.2) | 6,636 (14.9) | 1,440 (16.9) |  |
| 1-3 times/month | 6,637 (12.5) | 5,603 (12.5) | 1,034 (12.1) |  |
| 1-2 times/week | 12,650 (23.8) | 10,835 (24.3) | 1,815 (21.3) |  |
| 3-4 times/week | 9,773 (18.4) | 8,398 (18.8) | 1,375 (16.1) |  |
| Daily or almost daily | 9,718 (18.3) | 8,095 (18.1) | 1,623 (19.1) |  |
| Sleep duration |  |  |  | <0.001 |
| 7-8 hours/day | 35,436 (66.6) | 30107 (67.4) | 5,329 (62.6) |  |
| <7 or >8 hours/day | 17,735 (33.4) | 14548 (32.6) | 3,187 (37.4) |  |
| TyG index | 8.72 (8.34, 9.13) | 8.69 (8.32, 9.10) | 8.87 (8.49, 9.26) | <0.001 |
| TyG-BMI index | 239.18 (207.43, 276.96) | 236.26 (205.16, 273.57) | 254.77 (221.45, 293.29) | <0.001 |
| TyG-WC index | 786.87 (682.61, 898.43) | 775.50 (673.63, 885.68) | 849.00 (741.39, 956.06) | <0.001 |
| TyG-WHtR index | 4.71 (4.13, 5.35) | 4.65 (4.08, 5.27) | 5.05 (4.45, 5.66) | <0.001 |
| TyG-WWI index | 89.87 (82.35, 97.44) | 89.07 (81.66, 96.60) | 93.88 (86.55, 100.98) | <0.001 |
| TyG-ABSI index | 0.67 (0.61, 0.72) | 0.66 (0.61, 0.71) | 0.69 (0.64, 0.74) | <0.001 |
| TyG-BRI index | 35.83 (26.49, 47.26) | 34.79 (25.70, 45.92) | 41.61 (31.47, 53.56) | <0.001 |

*Note*: Data are presented as median (interquartile range) for continuous variables and *n* (%) for categorical variables.

*Abbreviations*: CVD, cardiovascular disease; TDI, Townsend deprivation index; TyG, triglyceride-glucose index; BMI, body mass index; WC, waist circumference; WHtR, weight-to-height ratio; BRI, body roundness index; ABSI, a body shape index; WWI, weight-adjusted waist index.

**Table S6. Sensitivity analysis for the associations between TyG-related indices and incident CVD and mortality in participants with depression: additionally adjusting for hypertension and diabetes.**

|  | **Total CVD**  **(*n* = 56,745)** | **All-cause mortality**  **(*n* = 56,745)** | **CVD mortality**  **(*n* = 56,745)** |
| --- | --- | --- | --- |
| **Exposures** | **HR (95% CI)** | **HR (95% CI)** | **HR (95% CI)** |
| **TyG index** |  |  |  |
| Per SD increment | 1.07 (1.04-1.09) | 0.98 (0.95-1.01) | 1.08 (1.00-1.18) |
| Quartile 1 | Reference | 1.10 (1.00-1.20) | Reference |
| Quartile 2 | 1.10 (1.03-1.18) | Reference | 1.20 (0.89-1.62) |
| Quartile 3 | 1.16 (1.09-1.24) | 1.03 (0.95-1.12) | 1.33 (1.00-1.76) |
| Quartile 4 | 1.21 (1.13-1.29) | 1.01 (0.94-1.10) | 1.35 (1.02-1.79) |
| **TyG-BMI index** |  |  |  |
| Per SD increment | 1.19 (1.16-1.21) | 1.00 (0.97-1.03) | 1.10 (1.01-1.20) |
| Quartile 1 | Reference | 1.22 (1.12-1.33) | Reference |
| Quartile 2 | 1.13 (1.05-1.21) | Reference | 1.04 (0.78-1.37) |
| Quartile 3 | 1.24 (1.16-1.33) | 0.98 (0.90-1.07) | 1.04 (0.79-1.37) |
| Quartile 4 | 1.47 (1.37-1.57) | 1.06 (0.97-1.15) | 1.16 (0.88-1.53) |
| **TyG-WC index** |  |  |  |
| Per SD increment | 1.22 (1.19-1.25) | 1.05 (1.02-1.09) | 1.18 (1.07-1.30) |
| Quartile 1 | Reference | 1.07 (0.97-1.17) | Reference |
| Quartile 2 | 1.17 (1.08-1.26) | Reference | 0.94 (0.68-1.29) |
| Quartile 3 | 1.35 (1.25-1.45) | 1.04 (0.95-1.13) | 1.17 (0.87-1.58) |
| Quartile 4 | 1.59 (1.47-1.71) | 1.11 (1.01-1.20) | 1.21 (0.89-1.65) |
| **TyG-WHtR index** |  |  |  |
| Per SD increment | 1.20 (1.17-1.23) | 1.05 (1.02-1.09) | 1.20 (1.09-1.32) |
| Quartile 1 | Reference | 1.07 (0.97-1.17) | Reference |
| Quartile 2 | 1.16 (1.08-1.25) | Reference | 0.92 (0.68-1.26) |
| Quartile 3 | 1.30 (1.21-1.39) | 1.04 (0.95-1.13) | 0.93 (0.69-1.25) |
| Quartile 4 | 1.55 (1.44-1.66) | 1.11 (1.01-1.20) | 1.31 (0.98-1.75) |
| **TyG-WWI index** |  |  |  |
| Per SD increment | 1.15 (1.12-1.17) | 1.09 (1.05-1.12) | 1.24 (1.13-1.37) |
| Quartile 1 | Reference | Reference | Reference |
| Quartile 2 | 1.23 (1.14-1.33) | 1.07 (0.97-1.18) | 1.11 (0.79-1.56) |
| Quartile 3 | 1.31 (1.22-1.41) | 1.08 (0.98-1.20) | 1.23 (0.89-1.70) |
| Quartile 4 | 1.48 (1.37-1.59) | 1.20 (1.09-1.32) | 1.58 (1.16-2.17) |
| **TyG-BRI index** |  |  |  |
| Per SD increment | 1.19 (1.17-1.22) | 1.08 (1.05-1.11) | 1.20 (1.10-1.31) |
| Quartile 1 | Reference | 1.07 (0.97-1.18) | Reference |
| Quartile 2 | 1.16 (1.08-1.25) | Reference | 0.98 (0.72-1.33) |
| Quartile 3 | 1.31 (1.22-1.41) | 0.95 (0.88-1.04) | 1.00 (0.74-1.34) |
| Quartile 4 | 1.58 (1.47-1.70) | 1.13 (1.04-1.22) | 1.25 (0.94-1.67) |
| **TyG-ABSI index** |  |  |  |
| Per SD increment | 1.11 (1.09-1.14) | 1.10 (1.07-1.14) | 1.23 (1.12-1.36) |
| Quartile 1 | Reference | Reference | Reference |
| Quartile 2 | 1.19 (1.11-1.28) | 1.12 (1.01-1.24) | 1.44 (1.01-2.04) |
| Quartile 3 | 1.25 (1.16-1.35) | 1.15 (1.04-1.27) | 1.37 (0.97-1.94) |
| Quartile 4 | 1.38 (1.28-1.49) | 1.27 (1.15-1.41) | 1.85 (1.31-2.61) |

Models were adjusted for age, sex, race, employment status, educational level, Townsend deprivation index, smoking status, drinking frequency, sleep duration, physical activity, hypertension, and diabetes.

Abbreviation: CVD, cardiovascular disease; SD, standard deviation; HR, hazard ratio; CI, confidence interval; TyG, triglyceride-glucose; BMI, body mass index; WC, waist circumference; WHtR, waist-to-height ratio; BRI, body roundness index; ABSI, a body shape index; WWI, weight-adjusted waist index.

**Table S7. Crude associations of TyG-related indices with incident CVD, CVD mortality, and all-cause mortality.**

|  | **Total CVD** | **All-cause mortality** | **CVD mortality** |
| --- | --- | --- | --- |
| **Exposures** | **HR (95% CI)** | **HR (95% CI)** | **HR (95% CI)** |
| **TyG index** |  |  |  |
| Per SD increment | 1.30 (1.28-1.33) | 1.22 (1.19-1.26) | 1.43 (1.33-1.55) |
| Quartile 1 | Reference | Reference | Reference |
| Quartile 2 | 1.43 (1.33-1.53) | 1.22 (1.12-1.34) | 1.71 (1.27-2.29) |
| Quartile 3 | 1.73 (1.62-1.84) | 1.46 (1.34-1.59) | 2.28 (1.72-3.02) |
| Quartile 4 | 2.08 (1.95-2.22) | 1.70 (1.57-1.85) | 2.96 (2.26-3.89) |
| **TyG-BMI index** |  |  |  |
| Per SD increment | 1.33 (1.30-1.35) | 1.16 (1.13-1.20) | 1.33 (1.24-1.43) |
| Quartile 1 | Reference | Reference | Reference |
| Quartile 2 | 1.44 (1.34-1.54) | 1.05 (0.97-1.15) | 1.48 (1.12-1.95) |
| Quartile 3 | 1.78 (1.67-1.91) | 1.17 (1.08-1.28) | 1.80 (1.37-2.35) |
| Quartile 4 | 2.31 (2.16-2.46) | 1.44 (1.32-1.56) | 2.36 (1.83-3.05) |
| **TyG-WC index** |  |  |  |
| Per SD increment | 1.48 (1.45-1.51) | 1.35 (1.32-1.39) | 1.65 (1.53-1.78) |
| Quartile 1 | Reference | Reference | Reference |
| Quartile 2 | 1.52 (1.41-1.64) | 1.26 (1.15-1.38) | 1.45 (1.05-1.99) |
| Quartile 3 | 2.10 (1.96-2.25) | 1.61 (1.47-1.76) | 2.50 (1.87-3.34) |
| Quartile 4 | 2.97 (2.78-3.18) | 2.16 (1.99-2.36) | 3.66 (2.78-4.82) |
| **TyG-WHtR index** |  |  |  |
| Per SD increment | 1.45 (1.42-1.48) | 1.34 (1.30-1.38) | 1.60 (1.48-1.72) |
| Quartile 1 | Reference | Reference | Reference |
| Quartile 2 | 1.55 (1.44-1.67) | 1.29 (1.18-1.42) | 1.45 (1.07-1.96) |
| Quartile 3 | 2.08 (1.94-2.23) | 1.54 (1.41-1.69) | 1.97 (1.47-2.63) |
| Quartile 4 | 2.90 (2.72-3.10) | 2.12 (1.95-2.31) | 3.56 (2.73-4.64) |
| **TyG-WWI index** |  |  |  |
| Per SD increment | 1.48 (1.45-1.51) | 1.46 (1.42-1.50) | 1.79 (1.65-1.93) |
| Quartile 1 | Reference | Reference | Reference |
| Quartile 2 | 1.66 (1.55-1.79) | 1.52 (1.38-1.68) | 1.79 (1.28-2.50) |
| Quartile 3 | 2.17 (2.02-2.33) | 1.93 (1.75-2.12) | 2.72 (1.99-3.72) |
| Quartile 4 | 3.05 (2.85-3.26) | 2.77 (2.53-3.03) | 4.93 (3.67-6.61) |
| **TyG-BRI index** |  |  |  |
| Per SD increment | 1.40 (1.37-1.42) | 1.31 (1.28-1.34) | 1.50 (1.41-1.60) |
| Quartile 1 | Reference | Reference | Reference |
| Quartile 2 | 1.53 (1.42-1.64) | 1.25 (1.14-1.37) | 1.50 (1.11-2.03) |
| Quartile 3 | 2.06 (1.92-2.21) | 1.47 (1.35-1.61) | 2.10 (1.58-2.80) |
| Quartile 4 | 2.88 (2.69-3.08) | 2.09 (1.92-2.28) | 3.35 (2.56-4.38) |
| **TyG-ABSI index** |  |  |  |
| Per SD increment | 1.45 (1.42-1.48) | 1.49 (1.45-1.53) | 1.82 (1.69-1.97) |
| Quartile 1 | Reference | Reference | Reference |
| Quartile 2 | 1.60 (1.49-1.72) | 1.57 (1.42-1.74) | 2.30 (1.62-3.25) |
| Quartile 3 | 2.07 (1.93-2.22) | 2.01 (1.83-2.22) | 3.05 (2.18-4.25) |
| Quartile 4 | 2.87 (2.68-3.06) | 2.93 (2.68-3.21) | 5.96 (4.35-8.15) |

Models were unadjusted for baseline covariates.

Abbreviation: CVD, cardiovascular disease; SD, standard deviation; HR, hazard ratio; CI, confidence interval; TyG, triglyceride-glucose; BMI, body mass index; WC, waist circumference; WHtR, waist-to-height ratio; BRI, body roundness index; ABSI, a body shape index; WWI, weight-adjusted waist index.

**Table S8. Associations between TyG-related indices and selected biomarkers in individuals with depression**

| **TyG-related indices** | **Biomarkers** | **Beta** | **95% Lower CI** | **95% Upper CI** | **FDR-P** | ***N*** |
| --- | --- | --- | --- | --- | --- | --- |
| TyG index |  |  |  |  |  |  |
|  | ALB | 0.036 | 0.027 | 0.045 | <0.001 | 53145 |
|  | ALT | 0.252 | 0.243 | 0.261 | <0.001 | 53148 |
|  | AST | 0.116 | 0.106 | 0.125 | <0.001 | 52923 |
|  | ALP | 0.151 | 0.142 | 0.160 | <0.001 | 53156 |
|  | DB | -0.141 | -0.150 | -0.131 | <0.001 | 42046 |
|  | GGT | 0.232 | 0.222 | 0.242 | <0.001 | 53123 |
|  | Total bilirubin | -0.123 | -0.130 | -0.115 | <0.001 | 52923 |
|  | Urea | 0.080 | 0.071 | 0.088 | <0.001 | 53141 |
|  | Creatinine | 0.006 | -0.001 | 0.013 | 0.122 | 53168 |
|  | Cystatin C | 0.130 | 0.122 | 0.138 | <0.001 | 53146 |
|  | Phosphate | 0.002 | -0.007 | 0.010 | 0.701 | 53075 |
|  | Total protein | 0.058 | 0.049 | 0.066 | <0.001 | 53091 |
|  | Urate | 0.255 | 0.248 | 0.263 | <0.001 | 53101 |
|  | Apolipoprotein A | -0.207 | -0.215 | -0.200 | <0.001 | 52834 |
|  | Apolipoprotein B | 0.337 | 0.328 | 0.345 | <0.001 | 52874 |
|  | Cholesterol | 0.266 | 0.257 | 0.274 | <0.001 | 53154 |
|  | HbA1c | 0.329 | 0.320 | 0.337 | <0.001 | 50368 |
|  | HDL cholesterol | -0.390 | -0.397 | -0.383 | <0.001 | 53159 |
|  | LDL direct | 0.265 | 0.256 | 0.274 | <0.001 | 53089 |
|  | Lipoprotein A | -0.038 | -0.048 | -0.028 | <0.001 | 42610 |
|  | CRP | 0.134 | 0.124 | 0.144 | <0.001 | 53051 |
|  | WBC | 0.163 | 0.155 | 0.172 | <0.001 | 51781 |
|  | Lymphocyte count | 0.103 | 0.096 | 0.111 | <0.001 | 51659 |
|  | Monocyte count | 0.053 | 0.047 | 0.060 | <0.001 | 51659 |
|  | Neutrophil count | 0.140 | 0.131 | 0.149 | <0.001 | 51659 |
|  | Eosinophil count | 0.078 | 0.069 | 0.087 | <0.001 | 51659 |
|  | Basophil count | 0.037 | 0.027 | 0.046 | <0.001 | 51659 |
|  | Platelet count | 0.087 | 0.078 | 0.096 | <0.001 | 51783 |
|  | Platelet crit | 0.092 | 0.083 | 0.101 | <0.001 | 51783 |
| TyG-BMI index |  |  |  |  |  |  |
|  | ALB | -0.137 | -0.146 | -0.129 | <0.001 | 53145 |
|  | ALT | 0.273 | 0.264 | 0.282 | <0.001 | 53148 |
|  | AST | 0.108 | 0.099 | 0.117 | <0.001 | 52923 |
|  | ALP | 0.163 | 0.155 | 0.172 | <0.001 | 53156 |
|  | DB | -0.078 | -0.087 | -0.069 | <0.001 | 42046 |
|  | GGT | 0.212 | 0.203 | 0.222 | <0.001 | 53123 |
|  | Total bilirubin | -0.095 | -0.103 | -0.087 | <0.001 | 52923 |
|  | Urea | 0.101 | 0.092 | 0.109 | <0.001 | 53141 |
|  | Creatinine | 0.046 | 0.039 | 0.054 | <0.001 | 53168 |
|  | Cystatin C | 0.240 | 0.233 | 0.248 | <0.001 | 53146 |
|  | Phosphate | -0.082 | -0.090 | -0.073 | <0.001 | 53075 |
|  | Total protein | 0.015 | 0.006 | 0.023 | <0.001 | 53091 |
|  | Urate | 0.381 | 0.375 | 0.388 | <0.001 | 53101 |
|  | Apolipoprotein A | -0.244 | -0.252 | -0.236 | <0.001 | 52834 |
|  | Apolipoprotein B | 0.167 | 0.158 | 0.175 | <0.001 | 52874 |
|  | Cholesterol | 0.055 | 0.047 | 0.064 | <0.001 | 53154 |
|  | HbA1c | 0.301 | 0.293 | 0.310 | <0.001 | 50368 |
|  | HDL cholesterol | -0.361 | -0.369 | -0.354 | <0.001 | 53159 |
|  | LDL direct | 0.098 | 0.089 | 0.107 | <0.001 | 53089 |
|  | Lipoprotein A | 0.002 | -0.008 | 0.012 | 0.675 | 42610 |
|  | CRP | 0.307 | 0.298 | 0.316 | <0.001 | 53051 |
|  | WBC | 0.174 | 0.165 | 0.182 | <0.001 | 51781 |
|  | Lymphocyte count | 0.102 | 0.094 | 0.110 | <0.001 | 51659 |
|  | Monocyte count | 0.079 | 0.072 | 0.086 | <0.001 | 51659 |
|  | Neutrophil count | 0.149 | 0.140 | 0.157 | <0.001 | 51659 |
|  | Eosinophil count | 0.102 | 0.093 | 0.111 | <0.001 | 51659 |
|  | Basophil count | 0.043 | 0.034 | 0.052 | <0.001 | 51659 |
|  | Platelet count | 0.081 | 0.072 | 0.090 | <0.001 | 51783 |
|  | Platelet crit | 0.097 | 0.088 | 0.106 | <0.001 | 51783 |
| TyG-WC index |  |  |  |  |  |  |
|  | ALB | -0.116 | -0.125 | -0.106 | <0.001 | 53145 |
|  | ALT | 0.317 | 0.307 | 0.326 | <0.001 | 53148 |
|  | AST | 0.131 | 0.121 | 0.141 | <0.001 | 52923 |
|  | ALP | 0.185 | 0.175 | 0.194 | <0.001 | 53156 |
|  | DB | -0.092 | -0.102 | -0.083 | <0.001 | 42046 |
|  | GGT | 0.254 | 0.244 | 0.265 | <0.001 | 53123 |
|  | Total bilirubin | -0.109 | -0.118 | -0.101 | <0.001 | 52923 |
|  | Urea | 0.103 | 0.094 | 0.112 | <0.001 | 53141 |
|  | Creatinine | 0.038 | 0.030 | 0.046 | <0.001 | 53168 |
|  | Cystatin C | 0.252 | 0.244 | 0.261 | <0.001 | 53146 |
|  | Phosphate | -0.071 | -0.080 | -0.061 | <0.001 | 53075 |
|  | Total protein | 0.031 | 0.022 | 0.041 | <0.001 | 53091 |
|  | Urate | 0.406 | 0.398 | 0.413 | <0.001 | 53101 |
|  | Apolipoprotein A | -0.277 | -0.286 | -0.269 | <0.001 | 52834 |
|  | Apolipoprotein B | 0.214 | 0.205 | 0.224 | <0.001 | 52874 |
|  | Cholesterol | 0.090 | 0.081 | 0.099 | <0.001 | 53154 |
|  | HbA1c | 0.356 | 0.347 | 0.366 | <0.001 | 50368 |
|  | HDL cholesterol | -0.421 | -0.429 | -0.414 | <0.001 | 53159 |
|  | LDL direct | 0.133 | 0.124 | 0.143 | <0.001 | 53089 |
|  | Lipoprotein A | -0.005 | -0.016 | 0.006 | 0.365 | 42610 |
|  | CRP | 0.307 | 0.297 | 0.317 | <0.001 | 53051 |
|  | WBC | 0.198 | 0.190 | 0.207 | <0.001 | 51781 |
|  | Lymphocyte count | 0.113 | 0.105 | 0.122 | <0.001 | 51659 |
|  | Monocyte count | 0.089 | 0.081 | 0.096 | <0.001 | 51659 |
|  | Neutrophil count | 0.172 | 0.163 | 0.182 | <0.001 | 51659 |
|  | Eosinophil count | 0.119 | 0.110 | 0.129 | <0.001 | 51659 |
|  | Basophil count | 0.046 | 0.036 | 0.056 | <0.001 | 51659 |
|  | Platelet count | 0.091 | 0.082 | 0.101 | <0.001 | 51783 |
|  | Platelet crit | 0.104 | 0.095 | 0.113 | <0.001 | 51783 |
| TyG-WHtR index |  |  |  |  |  |  |
|  | ALB | -0.110 | -0.119 | -0.101 | <0.001 | 53145 |
|  | ALT | 0.302 | 0.293 | 0.311 | <0.001 | 53148 |
|  | AST | 0.128 | 0.118 | 0.138 | <0.001 | 52923 |
|  | ALP | 0.185 | 0.176 | 0.194 | <0.001 | 53156 |
|  | DB | -0.095 | -0.104 | -0.085 | <0.001 | 42046 |
|  | GGT | 0.251 | 0.241 | 0.261 | <0.001 | 53123 |
|  | Total bilirubin | -0.114 | -0.122 | -0.106 | <0.001 | 52923 |
|  | Urea | 0.097 | 0.089 | 0.106 | <0.001 | 53141 |
|  | Creatinine | 0.026 | 0.018 | 0.033 | <0.001 | 53168 |
|  | Cystatin C | 0.235 | 0.227 | 0.243 | <0.001 | 53146 |
|  | Phosphate | -0.065 | -0.074 | -0.056 | <0.001 | 53075 |
|  | Total protein | 0.036 | 0.027 | 0.045 | <0.001 | 53091 |
|  | Urate | 0.387 | 0.380 | 0.394 | <0.001 | 53101 |
|  | Apolipoprotein A | -0.258 | -0.266 | -0.250 | <0.001 | 52834 |
|  | Apolipoprotein B | 0.212 | 0.203 | 0.221 | <0.001 | 52874 |
|  | Cholesterol | 0.093 | 0.084 | 0.101 | <0.001 | 53154 |
|  | HbA1c | 0.340 | 0.331 | 0.348 | <0.001 | 50368 |
|  | HDL cholesterol | -0.397 | -0.404 | -0.390 | <0.001 | 53159 |
|  | LDL direct | 0.133 | 0.124 | 0.142 | <0.001 | 53089 |
|  | Lipoprotein A | -0.005 | -0.015 | 0.005 | 0.351 | 42610 |
|  | CRP | 0.306 | 0.297 | 0.316 | <0.001 | 53051 |
|  | WBC | 0.207 | 0.199 | 0.216 | <0.001 | 51781 |
|  | Lymphocyte count | 0.115 | 0.107 | 0.123 | <0.001 | 51659 |
|  | Monocyte count | 0.091 | 0.084 | 0.098 | <0.001 | 51659 |
|  | Neutrophil count | 0.184 | 0.175 | 0.193 | <0.001 | 51659 |
|  | Eosinophil count | 0.117 | 0.108 | 0.126 | <0.001 | 51659 |
|  | Basophil count | 0.049 | 0.039 | 0.058 | <0.001 | 51659 |
|  | Platelet count | 0.109 | 0.100 | 0.119 | <0.001 | 51783 |
|  | Platelet crit | 0.120 | 0.111 | 0.129 | <0.001 | 51783 |
|  |  |  |  |  |  |  |
|  |  |  |  |  |  |  |
|  |  |  |  |  |  |  |
| **TyG-WWI index** |  |  |  |  |  |  |
|  | ALB | -0.031 | -0.040 | -0.022 | <0.001 | 53145 |
|  | ALT | 0.287 | 0.278 | 0.296 | <0.001 | 53148 |
|  | AST | 0.132 | 0.123 | 0.142 | <0.001 | 52923 |
|  | ALP | 0.183 | 0.174 | 0.192 | <0.001 | 53156 |
|  | DB | -0.117 | -0.127 | -0.108 | <0.001 | 42046 |
|  | GGT | 0.262 | 0.252 | 0.272 | <0.001 | 53123 |
|  | Total bilirubin | -0.126 | -0.135 | -0.118 | <0.001 | 52923 |
|  | Urea | 0.078 | 0.069 | 0.087 | <0.001 | 53141 |
|  | Creatinine | -0.006 | -0.013 | 0.002 | 0.161 | 53168 |
|  | Cystatin C | 0.178 | 0.170 | 0.186 | <0.001 | 53146 |
|  | Phosphate | -0.023 | -0.032 | -0.014 | <0.001 | 53075 |
|  | Total protein | 0.061 | 0.051 | 0.070 | <0.001 | 53091 |
|  | Urate | 0.319 | 0.311 | 0.326 | <0.001 | 53101 |
|  | Apolipoprotein A | -0.234 | -0.242 | -0.225 | <0.001 | 52834 |
|  | Apolipoprotein B | 0.277 | 0.268 | 0.286 | <0.001 | 52874 |
|  | Cholesterol | 0.176 | 0.167 | 0.185 | <0.001 | 53154 |
|  | HbA1c | 0.341 | 0.332 | 0.350 | <0.001 | 50368 |
|  | HDL cholesterol | -0.395 | -0.403 | -0.388 | <0.001 | 53159 |
|  | LDL direct | 0.198 | 0.189 | 0.207 | <0.001 | 53089 |
|  | Lipoprotein A | -0.020 | -0.031 | -0.010 | <0.001 | 42610 |
|  | CRP | 0.230 | 0.220 | 0.240 | <0.001 | 53051 |
|  | WBC | 0.211 | 0.202 | 0.220 | <0.001 | 51781 |
|  | Lymphocyte count | 0.114 | 0.105 | 0.122 | <0.001 | 51659 |
|  | Monocyte count | 0.084 | 0.077 | 0.091 | <0.001 | 51659 |
|  | Neutrophil count | 0.192 | 0.183 | 0.202 | <0.001 | 51659 |
|  | Eosinophil count | 0.111 | 0.102 | 0.120 | <0.001 | 51659 |
|  | Basophil count | 0.047 | 0.038 | 0.057 | <0.001 | 51659 |
|  | Platelet count | 0.125 | 0.115 | 0.134 | <0.001 | 51783 |
|  | Platelet crit | 0.127 | 0.118 | 0.137 | <0.001 | 51783 |
| **TyG-ABSI index** |  |  |  |  |  |  |
|  | ALB | 0.017 | 0.008 | 0.027 | <0.001 | 53145 |
|  | ALT | 0.261 | 0.251 | 0.271 | <0.001 | 53148 |
|  | AST | 0.126 | 0.115 | 0.136 | <0.001 | 52923 |
|  | ALP | 0.165 | 0.156 | 0.175 | <0.001 | 53156 |
|  | DB | -0.119 | -0.129 | -0.109 | <0.001 | 42046 |
|  | GGT | 0.248 | 0.237 | 0.259 | <0.001 | 53123 |
|  | Total bilirubin | -0.118 | -0.126 | -0.109 | <0.001 | 52923 |
|  | Urea | 0.063 | 0.053 | 0.072 | <0.001 | 53141 |
|  | Creatinine | -0.016 | -0.024 | -0.007 | <0.001 | 53168 |
|  | Cystatin C | 0.141 | 0.132 | 0.150 | <0.001 | 53146 |
|  | Phosphate | 0.001 | -0.008 | 0.011 | 0.789 | 53075 |
|  | Total protein | 0.067 | 0.057 | 0.077 | <0.001 | 53091 |
|  | Urate | 0.260 | 0.252 | 0.268 | <0.001 | 53101 |
|  | Apolipoprotein A | -0.212 | -0.220 | -0.203 | <0.001 | 52834 |
|  | Apolipoprotein B | 0.295 | 0.285 | 0.305 | <0.001 | 52874 |
|  | Cholesterol | 0.212 | 0.202 | 0.221 | <0.001 | 53154 |
|  | HbA1c | 0.328 | 0.318 | 0.337 | <0.001 | 50368 |
|  | HDL cholesterol | -0.378 | -0.386 | -0.370 | <0.001 | 53159 |
|  | LDL direct | 0.221 | 0.212 | 0.231 | <0.001 | 53089 |
|  | Lipoprotein A | -0.029 | -0.040 | -0.018 | <0.001 | 42610 |
|  | CRP | 0.163 | 0.152 | 0.174 | <0.001 | 53051 |
|  | WBC | 0.188 | 0.178 | 0.197 | <0.001 | 51781 |
|  | Lymphocyte count | 0.100 | 0.092 | 0.109 | <0.001 | 51659 |
|  | Monocyte count | 0.069 | 0.062 | 0.077 | <0.001 | 51659 |
|  | Neutrophil count | 0.173 | 0.163 | 0.183 | <0.001 | 51659 |
|  | Eosinophil count | 0.099 | 0.089 | 0.109 | <0.001 | 51659 |
|  | Basophil count | 0.041 | 0.030 | 0.051 | <0.001 | 51659 |
|  | Platelet count | 0.108 | 0.098 | 0.118 | <0.001 | 51783 |
|  | Platelet crit | 0.106 | 0.096 | 0.116 | <0.001 | 51783 |
| **TyG-BRI index** |  |  |  |  |  |  |
|  | ALB | -0.139 | -0.148 | -0.130 | <0.001 | 53145 |
|  | ALT | 0.273 | 0.264 | 0.282 | <0.001 | 53148 |
|  | AST | 0.117 | 0.107 | 0.126 | <0.001 | 52923 |
|  | ALP | 0.166 | 0.157 | 0.175 | <0.001 | 53156 |
|  | DB | -0.067 | -0.076 | -0.057 | <0.001 | 42046 |
|  | GGT | 0.223 | 0.213 | 0.233 | <0.001 | 53123 |
|  | Total bilirubin | -0.095 | -0.103 | -0.087 | <0.001 | 52923 |
|  | Urea | 0.089 | 0.081 | 0.098 | <0.001 | 53141 |
|  | Creatinine | 0.026 | 0.019 | 0.034 | <0.001 | 53168 |
|  | Cystatin C | 0.237 | 0.229 | 0.245 | <0.001 | 53146 |
|  | Phosphate | -0.075 | -0.084 | -0.066 | <0.001 | 53075 |
|  | Total protein | 0.023 | 0.014 | 0.031 | <0.001 | 53091 |
|  | Urate | 0.370 | 0.363 | 0.377 | <0.001 | 53101 |
|  | Apolipoprotein A | -0.232 | -0.240 | -0.224 | <0.001 | 52834 |
|  | Apolipoprotein B | 0.138 | 0.129 | 0.147 | <0.001 | 52874 |
|  | Cholesterol | 0.023 | 0.014 | 0.032 | <0.001 | 53154 |
|  | HbA1c | 0.309 | 0.300 | 0.318 | <0.001 | 50368 |
|  | HDL cholesterol | -0.339 | -0.347 | -0.332 | <0.001 | 53159 |
|  | LDL direct | 0.066 | 0.057 | 0.075 | <0.001 | 53089 |
|  | Lipoprotein A | 0.004 | -0.006 | 0.014 | 0.460 | 42610 |
|  | CRP | 0.320 | 0.310 | 0.329 | <0.001 | 53051 |
|  | WBC | 0.194 | 0.185 | 0.202 | <0.001 | 51781 |
|  | Lymphocyte count | 0.102 | 0.094 | 0.109 | <0.001 | 51659 |
|  | Monocyte count | 0.090 | 0.083 | 0.096 | <0.001 | 51659 |
|  | Neutrophil count | 0.175 | 0.166 | 0.184 | <0.001 | 51659 |
|  | Eosinophil count | 0.112 | 0.103 | 0.121 | <0.001 | 51659 |
|  | Basophil count | 0.047 | 0.038 | 0.057 | <0.001 | 51659 |
|  | Platelet count | 0.097 | 0.088 | 0.107 | <0.001 | 51783 |
|  | Platelet crit | 0.109 | 0.100 | 0.118 | <0.001 | 51783 |

*Note*: Associations between TyG-related indices and biomarkers were estimated using linear regression model, with results representing as beta and 95% CI. Models were adjusted for age, sex, race, employment status, educational level, Townsend deprivation index, smoking status, drinking frequency, sleep duration, and physical activity. Multiple comparisons were adjusted using FDR-*P* values.

*Abbreviation*: TyG, triglyceride-glucose; BMI, body mass index; WC, waist circumference; WHtR, waist-to-height ratio; BRI, body roundness index; ABSI, body shape index; WWI, weight-adjusted waist index; FDR, false discovery ratio; ALB, Albumin; ALT, Alanine aminotransferase; AST, Aspartate aminotransferase; DB, Direct bilirubin; GGT, Gamma glutamyltransferase; ALP, Alkaline phosphatase; HbA1c, Glycated hemoglobin; CRP, C-reactive protein; WBC, White blood cell count.

**Table S9. Associations between selected biomarkers and risk of cardiovascular disease in individuals with depression**

| **Classification** | **Biomarkers** | **HR** | **95% Lower CI** | **95% Upper CI** | ***P* value** | **FDR-*P*** | **Cases** |
| --- | --- | --- | --- | --- | --- | --- | --- |
| Liver function | ALB | 0.893 | 0.873 | 0.912 | <0.001 | <0.001 | 8513 |
|  | ALT | 1.060 | 1.042 | 1.078 | <0.001 | <0.001 | 8511 |
|  | AST | 1.058 | 1.043 | 1.074 | <0.001 | <0.001 | 8475 |
|  | ALP | 1.090 | 1.073 | 1.108 | <0.001 | <0.001 | 8516 |
|  | DB | 1.027 | 1.000 | 1.054 | 0.050 | 0.057 | 6831 |
|  | GGT | 1.081 | 1.068 | 1.095 | <0.001 | <0.001 | 8508 |
|  | Total bilirubin | 0.977 | 0.953 | 1.001 | 0.061 | 0.068 | 8473 |
|  | Total protein | 1.007 | 0.986 | 1.029 | 0.517 | 0.551 | 8506 |
| Renal function | Urea | 1.067 | 1.046 | 1.089 | <0.001 | <0.001 | 8511 |
|  | Creatinine | 1.065 | 1.047 | 1.083 | <0.001 | <0.001 | 8515 |
|  | Cystatin C | 1.127 | 1.116 | 1.139 | <0.001 | <0.001 | 8511 |
|  | Phosphate | 1.004 | 0.982 | 1.027 | 0.705 | 0.068 | 8502 |
|  | Urate | 1.174 | 1.147 | 1.202 | <0.001 | <0.001 | 8503 |
| Metabolic | Apolipoprotein A | 0.894 | 0.872 | 0.916 | <0.001 | <0.001 | 8457 |
|  | Apolipoprotein B | 0.996 | 0.976 | 1.017 | 0.729 | 0.729 | 8456 |
|  | Cholesterol | 0.955 | 0.935 | 0.975 | <0.001 | <0.001 | 8514 |
|  | HbA1c | 1.117 | 1.100 | 1.135 | <0.001 | <0.001 | 8093 |
|  | HDL cholesterol | 0.870 | 0.848 | 0.893 | <0.001 | <0.001 | 8515 |
|  | LDL direct | 0.970 | 0.950 | 0.991 | 0.005 | 0.006 | 8504 |
|  | Lipoprotein A | 1.051 | 1.027 | 1.076 | <0.001 | <0.001 | 6735 |
| Inflammatory | CRP | 1.100 | 1.085 | 1.116 | <0.001 | <0.001 | 8496 |
|  | WBC | 1.078 | 1.066 | 1.090 | <0.001 | <0.001 | 8298 |
|  | Lymphocyte count | 1.021 | 1.006 | 1.036 | 0.005 | 0.007 | 8283 |
|  | Monocyte count | 1.073 | 1.055 | 1.090 | <0.001 | <0.001 | 8283 |
|  | Neutrophil count | 1.144 | 1.121 | 1.167 | <0.001 | <0.001 | 8283 |
|  | Eosinophil count | 1.072 | 1.051 | 1.094 | <0.001 | <0.001 | 8283 |
|  | Basophil count | 1.042 | 1.024 | 1.062 | <0.001 | <0.001 | 8283 |
|  | Platelet count | 1.028 | 1.007 | 1.050 | 0.009 | 0.012 | 8298 |
|  | Platelet crit | 1.036 | 1.014 | 1.058 | 0.001 | 0.012 | 8298 |

*Note*: Associations between biomarkers and incident cardiovascular disease were estimated using Cox regression model, with results representing as HR and 95% CI. Models were adjusted for age, sex, race, employment status, educational level, Townsend deprivation index, smoking status, drinking frequency, sleep duration, and physical activity. Multiple comparisons were adjusted using FDR-*P* values.

*Abbreviation*: FDR, false discovery ratio; ALB, Albumin; ALT, Alanine aminotransferase; AST, Aspartate aminotransferase; DB, Direct bilirubin; GGT, Gamma glutamyltransferase; ALP, Alkaline phosphatase; HbA1c, Glycated hemoglobin; CRP, C-reactive protein; WBC, White blood cell count.

**Table S10. Proportion mediated by selected biomarkers as potential mediators in the associations between TyG-related indices and incident CVD in participants with depression**

| **Potential mediators** | **Proportion mediated [(PM, %) (95% CI)]** | | | | | | |
| --- | --- | --- | --- | --- | --- | --- | --- |
|  | **TyG index** | **TyG-BMI index** | **TyG-WC index** | **TyG-WHtR index** | **TyG-ABSI index** | **TyG-BRI index** | **TyG-WWI index** |
| **Liver function biomarkers** | | | | | | | |
| ALB | - | 4.9 (3.6-6.4)^***^ | 4.0 (3.0-5.1)^***^ | 4.0 (3.0-5.2)^***^ | - | 4.8 (3.5-6.3)^***^ | 1.7 (1.0-2.4)^***^ |
| ALT | 7.4 (3.6-11.7)^***^ | 0.9 (-1.5-3.1) | -0.1 (-2.5-2.4) | 2.0 (-2.3-2.7) | 5.3 (2.4-8.3)^**^ | 0.9 (-1.4-3.1) | 2.8 (-0.1-5.4) |
| AST | 4.6 (3.1-6.8)^***^ | 1.9 (1.2-2.8)^***^ | 1.9 (1.1-2.9)^***^ | 2.0 (1.2-3.0)^***^ | 3.6 (2.3-5.2)^***^ | 1.9 (1.2-2.9)^***^ | 2.8 (1.7-4.1)^***^ |
| ALP | 9.1 (7.0-12.0)^***^ | 4.6 (3.5-6.0)^***^ | 4.6 (3.4-6.0)^***^ | 4.8 (3.5-6.3)^***^ | 7.4 (5.6-9.7)^***^ | 4.6 (3.5-5.9)^***^ | 6.3 (4.7-8.2)^***^ |
| GGT | 12.3 (9.4-15.9)^***^ | 5.0 (3.6-6.4)^***^ | 5.0 (3.5-6.5)^***^ | 5.2 (3.7-6.7)^***^ | 9.4 (7.3-12.1)^***^ | 5.1 (3.7-6.4)^***^ | 7.4 (5.5-9.5)^***^ |
| **Renal function biomarkers** | | | | | | | |
| Urea | 3.5 (1.9-5.2)^***^ | 1.6 (0.6-2.6)^***^ | 1.5 (0.5-2.4)^***^ | 1.5 (0.6-2.4)^***^ | 2.1 (1.1-3.1)^***^ | 1.5 (0.6-2.4)^***^ | 1.9 (0.9-2.9)^***^ |
| Creatinine | - | 1.0 (0.6-1.4)^***^ | 0.8 (0.4-1.1)^***^ | 0.6 (0.3-0.9)^***^ | - | 0.6 (0.3-0.9)^***^ | - |
| Cystatin C | 11.7 (9.2-15.7)^***^ | 10.4 (8.7-12.9)^***^ | 9.8 (8.1-12.1)^***^ | 9.8 (8.1-12.2)^***^ | 9.5 (7.6-12.6)^***^ | 10.2 (8.4-12.5)^***^ | 9.7 (7.9-12.4)^***^ |
| Urate | 25.8 (19.9-33.7) ^***^ | 9.2 (4.9-13.4)^***^ | 8.6 (4.4-12.6)^***^ | 9.0 (4.9-13.0)^***^ | 19.0 (14.6-24.3)^***^ | 9.2 (5.2-13.0)^***^ | 15.4 (11.4-19.9)^***^ |
| **Metabolic biomarkers** | | | | | | | |
| Apolipoprotein A | 13.3 (8.6-19.1)^***^ | 4.2 (1.5-6.9)^***^ | 3.4 (0.6-6.2) | 3.8 (1.0-6.5)* | 9.6 (6.1-13.9)^***^ | 4.2 (1.7-6.8)^***^ | 6.8 (3.5-10.1)^***^ |
| HbA1c | 23.9 (17.5-31.8)^***^ | 7.2 (4.9-9.7)^***^ | 6.5 (4.0-9.1)^***^ | 6.9 (4.3-9.5)^***^ | 16.7 (12.3-21.4)^***^ | 7.3 (5.0-9.9)^***^ | 11.4 (8.0-14.9)^***^ |
| HDL-C | 27.4 (17.3-39.1)^***^ | 4.3 (-0.1-8.4) | 1.9 (-2.9-6.3) | 2.6 (-2.0-7.1) | 18.1 (11.1-25.9)^***^ | 4.9 (1.1-8.8)^*^ | 9.7 (4.0-15.3)^***^ |
| **Inflammatory biomarkers** | | | | | | | |
| CRP | 9.2 (7.2-11.7)^***^ | 8.0 (5.9-10.2)^***^ | 7.5 (5.7-9.4)^***^ | 7.8 (5.9-9.8)^***^ | 8.3 (6.6-10.3)^***^ | 8.0 (5.9-10.1)^***^ | 8.8 (7.1-10.9)^***^ |
| WBC | 8.9 (6.4-14.9)^***^ | 4.6 (3.3-7.5)^***^ | 4.7 (3.3-7.4)^***^ | 5.1 (3.6-8.0)^***^ | 7.6 (5.5-12.4)^***^ | 5.0 (3.5-7.8)^***^ | 6.7 (4.8-10.5)^***^ |
| Lymphocyte count | 1.2 (-0.4-3.1) | 0.3 (-0.8-1.2) | 0.2 (-1.0-1.1) | 0.2 (-1.2-1.2) | 0.8 (-0.3-2.2) | 0.3 (-0.8-1.2) | 0.5 (-0.8-1.7) |
| Monocyte count | 2.7 (1.8-4.1)^***^ | 1.7 (1.1-2.7)^***^ | 1.7 (1.0-2.7)^***^ | 1.9 (1.1-2.9)^***^ | 2.5 (1.6-3.8)^***^ | 1.9 (1.2-3.0)^***^ | 2.3 (1.5-3.6)^***^ |
| Neutrophil count | 13.1 (10.2-16.6)^***^ | 6.5 (5.2-7.9)^***^ | 6.6 (5.2-8.0)^***^ | 7.3 (5.6-8.9)^***^ | 11.6 (9.1-14.6)^***^ | 7.1 (5.6-8.7)^***^ | 9.9 (7.8-12.3)^***^ |
| Eosinophil count | 3.8 (2.5-5.3)^***^ | 2.0 (1.2-2.9)^***^ | 2.0 (1.1-2.9)^***^ | 2.1 (1.2-3.1)^***^ | 3.4 (2.2-4.7)^***^ | 2.1 (1.2-3.1)^***^ | 2.8 (1.7-4.0)^***^ |
| Basophil count | 1.1 (0.5-1.8)^***^ | 0.6 (0.2-0.9)* | 0.5 (0.2-0.9)^*^ | 0.6 (0.2-1.0)^*^ | 0.9 (0.4-1.4)^***^ | 0.6 (0.2-1.0)^*^ | 0.8 (0.3-1.2)^**^ |
| Platelet count | 1.6 (0.1-3.1) | 0.7 (-0.1-1.4) | 0.7 (-0.1-1.4) | 0.6 (-0.3-1.5) | 1.2 (-0.2-2.6) | 0.6 (-0.2-1.4) | 0.8 (-0.5-2.1) |
| Platelet crit | 2.1 (0.5-3.7)^*^ | 0.9 (0.1-1.7) | 0.8 (0.1-1.7) | 0.8 (-0.2-1.8) | 1.6 (0.3-3.0)^*^ | 0.8 (-0.1-1.8) | 1.1 (-0.2-2.5) |

*Note*: Models were adjusted for age, sex, race, employment status, educational level, Townsend deprivation index, smoking status, drinking frequency, sleep duration, and physical activity. ^***^FDR-adjusted *P* < 0.001; ^**^FDR-adjusted *P* < 0.01; ^*^FDR-adjusted *P* < 0.05.

Abbreviation: ALB, albumin; ALT, alanine aminotransferase; AST, aspartate aminotransferase; ALP, alkaline phosphatase GGT, gamma glutamyltransferas; CRP, C-reactive protein; HbA1c, glycated hemoglobin; WBC, white blood cell count; TyG, triglyceride-glucose; BMI, body mass index; WC, waist circumference; WHtR, waist-to-height ratio; BRI, body roundness index; ABSI, a body shape index; WWI, weight-adjusted waist index.

**Table S11. Sensitivity analysis for the associations between TyG-related indices and incident CVD and mortality in participants with depression: excluding participants who developed outcomes within the first 2 years of follow-up**

|  | **Total CVD**  **(*n* = 52,324)** | **All-cause mortality**  **(*n* = 52,917)** | **CVD mortality**  **(*n* = 53,149)** |
| --- | --- | --- | --- |
| **Exposures** | **HR (95% CI)** | **HR (95% CI)** | **HR (95% CI)** |
| **TyG index** |  |  |  |
| Per SD increment | 1.14 (1.11-1.17) | 1.05 (1.01-1.08) | 1.18 (1.09-1.29) |
| Quartile 1 | Reference | 1.07 (0.97-1.17) | Reference |
| Quartile 2 | 1.16 (1.08-1.25) | Reference | 1.27 (0.94-1.72) |
| Quartile 3 | 1.27 (1.19-1.36) | 1.06 (0.97-1.15) | 1.47 (1.10-1.96) |
| Quartile 4 | 1.41 (1.32-1.52) | 1.15 (1.06-1.25) | 1.63 (1.23-2.16) |
| **TyG-BMI index** |  |  |  |
| Per SD increment | 1.28 (1.26-1.31) | 1.09 (1.06-1.12) | 1.23 (1.13-1.34) |
| Quartile 1 | Reference | 1.16 (1.06-1.27) | Reference |
| Quartile 2 | 1.20 (1.11-1.29) | Reference | 1.09 (0.82-1.45) |
| Quartile 3 | 1.39 (1.29-1.49) | 1.04 (0.96-1.14) | 1.19 (0.91-1.57) |
| Quartile 4 | 1.82 (1.70-1.95) | 1.25 (1.15-1.36) | 1.57 (1.20-2.05) |
| **TyG-WC index** |  |  |  |
| Per SD increment | 1.32 (1.29-1.35) | 1.15 (1.11-1.19) | 1.31 (1.20-1.44) |
| Quartile 1 | Reference | 1.00 (0.91-1.11) | Reference |
| Quartile 2 | 1.24 (1.15-1.34) | Reference | 0.99 (0.71-1.37) |
| Quartile 3 | 1.50 (1.39-1.62) | 1.09 (1.00-1.19) | 1.33 (0.98-1.80) |
| Quartile 4 | 1.98 (1.84-2.14) | 1.30 (1.19-1.41) | 1.63 (1.21-2.20) |
| **TyG-WHtR index** |  |  |  |
| Per SD increment | 1.30 (1.27-1.33) | 1.15 (1.11-1.18) | 1.33 (1.22-1.45) |
| Quartile 1 | Reference | 1.01 (0.92-1.12) | Reference |
| Quartile 2 | 1.24 (1.14-1.34) | Reference | 0.93 (0.68-1.28) |
| Quartile 3 | 1.44 (1.33-1.55) | 1.02 (0.93-1.11) | 1.06 (0.78-1.43) |
| Quartile 4 | 1.92 (1.79-2.07) | 1.28 (1.18-1.39) | 1.71 (1.30-2.27) |
| **TyG-WWI index** |  |  |  |
| Per SD increment | 1.23 (1.20-1.26) | 1.17 (1.13-1.21) | 1.36 (1.24-1.49) |
| Quartile 1 | Reference | Reference | Reference |
| Quartile 2 | 1.28 (1.18-1.38) | 1.11 (1.01-1.24) | 1.17 (0.83-1.65) |
| Quartile 3 | 1.43 (1.32-1.54) | 1.18 (1.06-1.30) | 1.41 (1.02-1.96) |
| Quartile 4 | 1.77 (1.65-1.91) | 1.42 (1.29-1.57) | 2.02 (1.48-2.76) |
| **TyG-BRI index** |  |  |  |
| Per SD increment | 1.29 (1.26-1.31) | 1.16 (1.13-1.19) | 1.31 (1.21-1.42) |
| Quartile 1 | Reference | 1.03 (0.94-1.14) | Reference |
| Quartile 2 | 1.24 (1.15-1.34) | Reference | 1.00 (0.73-1.36) |
| Quartile 3 | 1.46 (1.36-1.58) | 1.02 (0.93-1.11) | 1.15 (0.85-1.54) |
| Quartile 4 | 1.95 (1.81-2.10) | 1.32 (1.21-1.43) | 1.66 (1.25-2.20) |
| **TyG-ABSI index** |  |  |  |
| Per SD increment | 1.19 (1.16-1.22) | 1.18 (1.14-1.22) | 1.33 (1.21-1.46) |
| Quartile 1 | Reference | Reference | Reference |
| Quartile 2 | 1.26 (1.16-1.36) | 1.15 (1.03-1.27) | 1.56 (1.09-2.23) |
| Quartile 3 | 1.36 (1.26-1.47) | 1.23 (1.11-1.36) | 1.53 (1.07-2.18) |
| Quartile 4 | 1.61 (1.49-1.74) | 1.47 (1.33-1.63) | 2.28 (1.61-3.22) |

Models were adjusted for age, sex, race, employment status, educational level, Townsend deprivation index, smoking status, drinking frequency, sleep duration, and physical activity.

Abbreviation: CVD, cardiovascular disease; SD, standard deviation; HR, hazard ratio; CI, confidence interval; TyG, triglyceride-glucose; BMI, body mass index; WC, waist circumference; WHtR, waist-to-height ratio; BRI, body roundness index; ABSI, a body shape index; WWI, weight-adjusted waist index.

**Table S12. Sensitivity analysis for the associations between TyG-related indices and incident CVD and mortality in participants with depression: imputing covariates with missing values using multiple imputation approach**

|  | **Total CVD**  **(*n* = 56,745)** | **All-cause mortality**  **(*n* = 56,745)** | **CVD mortality**  **(*n* = 56,745)** |
| --- | --- | --- | --- |
| **Exposures** | **HR (95% CI)** | **HR (95% CI)** | **HR (95% CI)** |
| **TyG index** |  |  |  |
| Per SD increment | 1.14 (1.12-1.17) | 1.04 (1.01-1.07) | 1.19 (1.10-1.29) |
| Quartile 1 | Reference | 1.07 (0.98-1.17) | Reference |
| Quartile 2 | 1.14 (1.07-1.22) | Reference | 1.29 (0.98-1.71) |
| Quartile 3 | 1.24 (1.17-1.32) | 1.05 (0.97-1.14) | 1.48 (1.13-1.93) |
| Quartile 4 | 1.40 (1.31-1.49) | 1.12 (1.04-1.21) | 1.66 (1.28-2.15) |
| **TyG-BMI index** |  |  |  |
| Per SD increment | 1.28 (1.26-1.31) | 1.08 (1.05-1.11) | 1.27 (1.18-1.37) |
| Quartile 1 | Reference | 1.21 (1.11-1.31) | Reference |
| Quartile 2 | 1.17 (1.10-1.25) | Reference | 1.10 (0.85-1.44) |
| Quartile 3 | 1.39 (1.30-1.48) | 1.06 (0.98-1.15) | 1.23 (0.95-1.58) |
| Quartile 4 | 1.80 (1.69-1.91) | 1.26 (1.17-1.36) | 1.65 (1.29-2.11) |
| **TyG-WC index** |  |  |  |
| Per SD increment | 1.32 (1.29-1.35) | 1.14 (1.10-1.17) | 1.35 (1.24-1.47) |
| Quartile 1 | Reference | 1.02 (0.93-1.11) | Reference |
| Quartile 2 | 1.22 (1.14-1.31) | Reference | 1.02 (0.75-1.38) |
| Quartile 3 | 1.52 (1.42-1.63) | 1.09 (1.00-1.18) | 1.38 (1.04-1.83) |
| Quartile 4 | 1.97 (1.84-2.11) | 1.25 (1.16-1.36) | 1.75 (1.33-2.31) |
| **TyG-WHtR index** |  |  |  |
| Per SD increment | 1.30 (1.27-1.33) | 1.14 (1.11-1.17) | 1.37 (1.27-1.48) |
| Quartile 1 | Reference | 1.02 (0.93-1.11) | Reference |
| Quartile 2 | 1.23 (1.14-1.32) | Reference | 0.91 (0.68-1.22) |
| Quartile 3 | 1.45 (1.36-1.55) | 1.09 (1.00-1.18) | 1.10 (0.84-1.45) |
| Quartile 4 | 1.92 (1.79-2.05) | 1.25 (1.16-1.36) | 1.71 (1.32-2.21) |
| **TyG-WWI index** |  |  |  |
| Per SD increment | 1.24 (1.21-1.27) | 1.16 (1.13-1.20) | 1.39 (1.27-1.51) |
| Quartile 1 | Reference | Reference | Reference |
| Quartile 2 | 1.31 (1.22-1.41) | 1.11 (1.01-1.24) | 1.26 (0.92-1.73) |
| Quartile 3 | 1.46 (1.36-1.56) | 1.18 (1.06-1.30) | 1.45 (1.07-1.97) |
| Quartile 4 | 1.80 (1.68-1.93) | 1.42 (1.29-1.57) | 2.11 (1.58-2.82) |
| **TyG-BRI index** |  |  |  |
| Per SD increment | 1.29 (1.26-1.31) | 1.15 (1.12-1.18) | 1.35 (1.26-1.45) |
| Quartile 1 | Reference | 1.05 (0.96-1.15) | Reference |
| Quartile 2 | 1.20 (1.12-1.28) | Reference | 1.04 (0.78-1.39) |
| Quartile 3 | 1.46 (1.36-1.56) | 1.02 (0.94-1.10) | 1.13 (0.86-1.48) |
| Quartile 4 | 1.93 (1.80-2.06) | 1.31 (1.21-1.41) | 1.72 (1.33-2.23) |
| **TyG-ABSI index** |  |  |  |
| Per SD increment | 1.19 (1.16-1.22) | 1.17 (1.13-1.21) | 1.34 (1.23-1.46) |
| Quartile 1 | Reference | Reference | Reference |
| Quartile 2 | 1.24 (1.16-1.33) | 1.16 (1.05-1.27) | 1.39 (1.01-1.92) |
| Quartile 3 | 1.38 (1.29-1.48) | 1.25 (1.14-1.38) | 1.45 (1.06-1.98) |
| Quartile 4 | 1.61 (1.50-1.73) | 1.45 (1.32-1.60) | 2.10 (1.54-2.85) |

Models were adjusted for age, sex, race, employment status, educational level, Townsend deprivation index, smoking status, drinking frequency, sleep duration, and physical activity.

Abbreviation: CVD, cardiovascular disease; SD, standard deviation; HR, hazard ratio; CI, confidence interval; TyG, triglyceride-glucose; BMI, body mass index; WC, waist circumference; WHtR, waist-to-height ratio; BRI, body roundness index; ABSI, a body shape index; WWI, weight-adjusted waist index.

**Table S13. Sensitivity analysis for the associations between TyG-related indices and incident CVD in participants with depression: using stricter definitions of baseline depression**

|  | **Analyses restricted to ICD-10-coded depression (*n* = 30,277)** | **Analyses restricted to PHQ-2-defined probable depression**  **(*n* = 40,968)** |
| --- | --- | --- |
| **Exposures** | **HR (95% CI)** | **HR (95% CI)** |
| **TyG index** |  |  |
| Per SD increment | 1.13 (1.10-1.16) | 1.16 (1.14-1.19) |
| Quartile 1 | Reference | Reference |
| Quartile 2 | 1.12 (1.02-1.22) | 1.12 (1.04-1.22) |
| Quartile 3 | 1.20 (1.10-1.31) | 1.26 (1.17-1.36) |
| Quartile 4 | 1.36 (1.24-1.48) | 1.46 (1.36-1.58) |
| **TyG-BMI index** |  |  |
| Per SD increment | 1.27 (1.23-1.30) | 1.30 (1.27-1.33) |
| Quartile 1 | Reference | Reference |
| Quartile 2 | 1.12 (1.02-1.23) | 1.32 (1.22-1.43) |
| Quartile 3 | 1.35 (1.24-1.48) | 1.49 (1.38-1.62) |
| Quartile 4 | 1.72 (1.57-1.88) | 1.94 (1.80-2.10) |
| **TyG-WC index** |  |  |
| Per SD increment | 1.31 (1.27-1.35) | 1.35 (1.31-1.38) |
| Quartile 1 | Reference | Reference |
| Quartile 2 | 1.21 (1.10-1.34) | 1.39 (1.27-1.51) |
| Quartile 3 | 1.52 (1.38-1.68) | 1.60 (1.47-1.74) |
| Quartile 4 | 1.88 (1.70-2.07) | 2.19 (2.01-2.38) |
| **TyG-WHtR index** |  |  |
| Per SD increment | 1.29 (1.25-1.32) | 1.32 (1.29-1.36) |
| Quartile 1 | Reference | Reference |
| Quartile 2 | 1.21 (1.10-1.34) | 1.33 (1.22-1.45) |
| Quartile 3 | 1.44 (1.31-1.58) | 1.56 (1.43-1.69) |
| Quartile 4 | 1.84 (1.68-2.02) | 2.08 (1.92-2.25) |
| **TyG-WWI index** |  |  |
| Per SD increment | 1.23 (1.19-1.27) | 1.26 (1.23-1.29) |
| Quartile 1 | Reference | Reference |
| Quartile 2 | 1.25 (1.13-1.38) | 1.32 (1.21-1.43) |
| Quartile 3 | 1.40 (1.27-1.54) | 1.49 (1.37-1.62) |
| Quartile 4 | 1.73 (1.57-1.90) | 1.87 (1.73-2.03) |
| **TyG-BRI index** |  |  |
| Per SD increment | 1.27 (1.24-1.31) | 1.30 (1.27-1.33) |
| Quartile 1 | Reference | Reference |
| Quartile 2 | 1.24 (1.12-1.36) | 1.25 (1.15-1.36) |
| Quartile 3 | 1.46 (1.32-1.60) | 1.43 (1.32-1.56) |
| Quartile 4 | 1.90 (1.73-2.09) | 1.73 (1.58-1.88) |
| **TyG-ABSI index** |  |  |
| Per SD increment | 1.19 (1.16-1.23) | 1.22 (1.18-1.25) |
| Quartile 1 | Reference | Reference |
| Quartile 2 | 1.21 (1.10-1.34) | 1.32 (1.21-1.44) |
| Quartile 3 | 1.35 (1.23-1.49) | 1.58 (1.45-1.71) |
| Quartile 4 | 1.61 (1.46-1.78) | 2.07 (1.91-2.24) |

Models were adjusted for age, sex, race, employment status, educational level, Townsend deprivation index, smoking status, drinking frequency, sleep duration, and physical activity.

Abbreviation: ICD-10, International Classification of Diseases, 10th Revision; PHQ-2, Patient Health Questionnaire-2; CVD, cardiovascular disease; SD, standard deviation; HR, hazard ratio; CI, confidence interval; TyG, triglyceride-glucose; BMI, body mass index; WC, waist circumference; WHtR, waist-to-height ratio; BRI, body roundness index; ABSI, a body shape index; WWI, weight-adjusted waist index.

**Table S14. Sensitivity analysis for the associations between TyG-related indices and incident CVD and mortality in participants with depression: using the Fine–Gray competing risk model.**

|  | **Total CVD incidence** | **CVD mortality** |
| --- | --- | --- |
| **Exposures** | **SHR (95% CI)** | **SHR (95% CI)** |
| **TyG index** |  |  |
| Per SD increment | 1.14 (1.12-1.16) | 1.20 (1.11-1.30) |
| Quartile 1 | Reference | Reference |
| Quartile 2 | 1.16 (1.08-1.24) | 1.30 (0.97-1.75) |
| Quartile 3 | 1.26 (1.18-1.35) | 1.51 (1.14-2.01) |
| Quartile 4 | 1.41 (1.32-1.51) | 1.73 (1.31-2.28) |
| **TyG-BMI index** |  |  |
| Per SD increment | 1.29 (1.26-1.32) | 1.28 (1.17-1.39) |
| Quartile 1 | Reference | Reference |
| Quartile 2 | 1.21 (1.13-1.30) | 1.19 (0.90-1.58) |
| Quartile 3 | 1.42 (1.32-1.52) | 1.33 (1.01-1.75) |
| Quartile 4 | 1.85 (1.73-1.98) | 1.78 (1.37-2.31) |
| **TyG-WC index** |  |  |
| Per SD increment | 1.32 (1.29-1.35) | 1.36 (1.24-1.50) |
| Quartile 1 | Reference | Reference |
| Quartile 2 | 1.24 (1.16-1.34) | 1.05 (0.76-1.45) |
| Quartile 3 | 1.52 (1.42-1.64) | 1.45 (1.08-1.94) |
| Quartile 4 | 1.98 (1.84-2.13) | 1.80 (1.34-2.41) |
| **TyG-WHtR index** |  |  |
| Per SD increment | 1.30 (1.27-1.33) | 1.37 (1.26-1.50) |
| Quartile 1 | Reference | Reference |
| Quartile 2 | 1.23 (1.14-1.32) | 1.02 (0.75-1.39) |
| Quartile 3 | 1.46 (1.36-1.57) | 1.15 (0.86-1.54) |
| Quartile 4 | 1.92 (1.79-2.06) | 1.89 (1.44-2.48) |
| **TyG-WWI index** |  |  |
| Per SD increment | 1.23 (1.20-1.26) | 1.38 (1.26-1.51) |
| Quartile 1 | Reference | Reference |
| Quartile 2 | 1.30 (1.20-1.39) | 1.19 (0.85-1.67) |
| Quartile 3 | 1.45 (1.35-1.56) | 1.42 (1.03-1.96) |
| Quartile 4 | 1.77 (1.65-1.90) | 2.07 (1.52-2.81) |
| **TyG-BRI index** |  |  |
| Per SD increment | 1.29 (1.26-1.31) | 1.35 (1.25-1.46) |
| Quartile 1 | Reference | Reference |
| Quartile 2 | 1.24 (1.15-1.33) | 1.09 (0.80-1.48) |
| Quartile 3 | 1.49 (1.38-1.60) | 1.25 (0.94-1.67) |
| Quartile 4 | 1.96 (1.83-2.10) | 1.83 (1.39-2.40) |
| **TyG-ABSI index** |  |  |
| Per SD increment | 1.18 (1.15-1.21) | 1.34 (1.22-1.47) |
| Quartile 1 | Reference | Reference |
| Quartile 2 | 1.24 (1.15-1.34) | 1.51 (1.06-2.14) |
| Quartile 3 | 1.36 (1.27-1.47) | 1.52 (1.08-2.15) |
| Quartile 4 | 1.59 (1.48-1.71) | 2.24 (1.60-3.14) |

*Note*: Fine-Gray subdistribution hazard models were used, with non-CVD death before incident CVD treated as a competing event. Models were adjusted for age, sex, race, Townsend Deprivation Index, educational attainment, employment status, smoking status, drinking frequency, physical activity, and sleep duration.

*Abbreviations*: CVD, cardiovascular disease; SD, standard deviation; SHR, subdistribution hazard ratio; CI, confidence interval; TyG, triglyceride-glucose; BMI, body mass index; WC, waist circumference; WHtR, waist-to-height ratio; BRI, body roundness index; ABSI, a body shape index; WWI, weight-adjusted waist index.

**Table S15. Subgroup analysis for association between TyG-related indices and incident CVD in individuals with depression**

| **Subgroups** | **Level** | ***N*** | **Cases** | **HR (95% CI) for Per SD increase** |
| --- | --- | --- | --- | --- |
| **TyG index** | | | | |
| Sex | Female | 34,939 | 4,600 | 1.21 (1.18-1.25) |
|  | Male | 18,232 | 3,916 | 1.10 (1.07-1.14) |
| Age (years) | ≥60 | 18,544 | 4,575 | 1.09 (1.06-1.13) |
|  | <60 | 34,627 | 3,941 | 1.22 (1.18-1.26) |
| Townsend deprivation index | <median | 26,570 | 3,936 | 1.14 (1.10-1.18) |
|  | ≥median | 26,601 | 4,580 | 1.17 (1.14-1.21) |
| Educational level | University or college | 15,988 | 2,060 | 1.18 (1.13-1.23) |
|  | Others | 37,183 | 6,456 | 1.15 (1.13-1.18) |
| Employed status | Employed | 44,532 | 6,943 | 1.15 (1.13-1.18) |
|  | Not employed | 8,639 | 1,573 | 1.17 (1.11-1.22) |
| Ethnicity | White | 49,940 | 8,086 | 1.15 (1.13-1.18) |
|  | Non-white | 3,231 | 430 | 1.25 (1.15-1.36) |
| Smoking status | Non-smoking | 25,980 | 4,778 | 1.14 (1.10-1.17) |
|  | Smoking | 27,191 | 3,738 | 1.19 (1.16-1.23) |
| Drinking frequency | ≥3 times/week | 19,491 | 2,998 | 1.13 (1.09-1.17) |
|  | <3 times/week | 33,680 | 5,518 | 1.18 (1.15-1.21) |
| Physical activity | Inadequate | 23,330 | 3,945 | 1.17 (1.13-1.20) |
|  | Adequate | 29,841 | 4,571 | 1.17 (1.13-1.20) |
| Sleep duration | 7-8 hours/day | 35,436 | 5,329 | 1.16 (1.13-1.20) |
|  | <7 or >8 hours/day | 17,735 | 3,187 | 1.17 (1.13-1.21) |
| **TyG-BMI** |  |  |  |  |
| Sex | Female | 34,939 | 4,600 | 1.29 (1.25-1.32) |
|  | Male | 18,232 | 3,916 | 1.28 (1.24-1.32) |
| Age (years) | ≥60 | 18,544 | 4,575 | 1.24 (1.20-1.27) |
|  | <60 | 34,627 | 3,941 | 1.32 (1.29-1.36) |
| Townsend deprivation index | <median | 26,570 | 3,936 | 1.29 (1.25-1.33) |
|  | ≥median | 26,601 | 4,580 | 1.28 (1.25-1.32) |
| Educational level | University or college | 15,988 | 2,060 | 1.33 (1.27-1.38) |
|  | Others | 37,183 | 6,456 | 1.27 (1.25-1.30) |
| Employed status | Employed | 44,532 | 6,943 | 1.30 (1.27-1.33) |
|  | Not employed | 8,639 | 1,573 | 1.25 (1.20-1.31) |
| Ethnicity | White | 49,940 | 8,086 | 1.28 (1.25-1.31) |
|  | Non-white | 3,231 | 430 | 1.45 (1.32-1.58) |
| Smoking status | Non-smoking | 25,980 | 4,778 | 1.24 (1.20-1.27) |
|  | Smoking | 27,191 | 3,738 | 1.36 (1.32-1.40) |
| Drinking frequency | ≥3 times/week | 19,491 | 2,998 | 1.27 (1.22-1.32) |
|  | <3 times/week | 33,680 | 5,518 | 1.29 (1.26-1.32) |
| Physical activity | Inadequate | 23,330 | 3,945 | 1.27 (1.24-1.31) |
|  | Adequate | 29,841 | 4,571 | 1.30 (1.26-1.34) |
| Sleep duration | 7-8 hours/day | 35,436 | 5,329 | 1.32 (1.28-1.35) |
|  | <7 or >8 hours/day | 17,735 | 3,187 | 1.24 (1.21-1.28) |
| **TyG-WC** |  |  |  |  |
| Sex | Female | 34,939 | 4,600 | 1.36 (1.32-1.41) |
|  | Male | 18,232 | 3,916 | 1.31 (1.26-1.35) |
| Age (years) | ≥60 | 18,544 | 4,575 | 1.27 (1.23-1.31) |
|  | <60 | 34,627 | 3,941 | 1.40 (1.36-1.44) |
| Townsend deprivation index | <median | 26,570 | 3,936 | 1.33 (1.28-1.38) |
|  | ≥median | 26,601 | 4,580 | 1.35 (1.31-1.39) |
| Educational level | University or college | 15,988 | 2,060 | 1.37 (1.31-1.44) |
|  | Others | 37,183 | 6,456 | 1.33 (1.30-1.37) |
| Employed status | Employed | 44,532 | 6,943 | 1.35 (1.31-1.38) |
|  | Not employed | 8,639 | 1,573 | 1.32 (1.26-1.38) |
| Ethnicity | White | 49,940 | 8,086 | 1.34 (1.30-1.37) |
|  | Non-white | 3,231 | 430 | 1.51 (1.36-1.68) |
| Smoking status | Non-smoking | 25,980 | 4,778 | 1.29 (1.25-1.33) |
|  | Smoking | 27,191 | 3,738 | 1.42 (1.37-1.47) |
| Drinking frequency | ≥3 times/week | 19,491 | 2,998 | 1.32 (1.27-1.38) |
|  | <3 times/week | 33,680 | 5,518 | 1.35 (1.31-1.39) |
| Physical activity | Inadequate | 23,330 | 3,945 | 1.33 (1.29-1.37) |
|  | Adequate | 29,841 | 4,571 | 1.36 (1.31-1.40) |
| Sleep duration | 7-8 hours/day | 35,436 | 5,329 | 1.37 (1.33-1.41) |
|  | <7 or >8 hours/day | 17,735 | 3,187 | 1.31 (1.26-1.35) |
| **TyG-WHtR** |  |  |  |  |
| Sex | Female | 34,939 | 4,600 | 1.33 (1.30-1.37) |
|  | Male | 18,232 | 3,916 | 1.31 (1.27-1.36) |
| Age (years) | ≥60 | 18,544 | 4,575 | 1.25 (1.21-1.29) |
|  | <60 | 34,627 | 3,941 | 1.39 (1.35-1.43) |
| Townsend deprivation index | <median | 26,570 | 3,936 | 1.30 (1.26-1.35) |
|  | ≥median | 26,601 | 4,580 | 1.35 (1.31-1.38) |
| Educational level | University or college | 15,988 | 2,060 | 1.35 (1.29-1.41) |
|  | Others | 37,183 | 6,456 | 1.32 (1.29-1.35) |
| Employed status | Employed | 44,532 | 6,943 | 1.33 (1.30-1.36) |
|  | Not employed | 8,639 | 1,573 | 1.31 (1.25-1.37) |
| Ethnicity | White | 49,940 | 8,086 | 1.32 (1.29-1.35) |
|  | Non-white | 3,231 | 430 | 1.51 (1.37-1.67) |
| Smoking status | Non-smoking | 25,980 | 4,778 | 1.29 (1.25-1.32) |
|  | Smoking | 27,191 | 3,738 | 1.39 (1.34-1.43) |
| Drinking frequency | ≥3 times/week | 19,491 | 2,998 | 1.30 (1.25-1.36) |
|  | <3 times/week | 33,680 | 5,518 | 1.34 (1.30-1.37) |
| Physical activity | Inadequate | 23,330 | 3,945 | 1.32 (1.28-1.36) |
|  | Adequate | 29,841 | 4,571 | 1.33 (1.29-1.38) |
| Sleep duration | 7-8 hours/day | 35,436 | 5,329 | 1.35 (1.31-1.39) |
|  | <7 or >8 hours/day | 17,735 | 3,187 | 1.30 (1.26-1.34) |
| **TyG-WWI** |  |  |  |  |
| Sex | Female | 34,939 | 4,600 | 1.30 (1.26-1.34) |
|  | Male | 18,232 | 3,916 | 1.25 (1.21-1.30) |
| Age (years) | ≥60 | 18,544 | 4,575 | 1.20 (1.16-1.24) |
|  | <60 | 34,627 | 3,941 | 1.37 (1.33-1.41) |
| Townsend deprivation index | <median | 26,570 | 3,936 | 1.24 (1.20-1.28) |
|  | ≥median | 26,601 | 4,580 | 1.32 (1.28-1.36) |
| Educational level | University or college | 15,988 | 2,060 | 1.30 (1.24-1.36) |
|  | Others | 37,183 | 6,456 | 1.28 (1.25-1.31) |
| Employed status | Employed | 44,532 | 6,943 | 1.28 (1.25-1.31) |
|  | Not employed | 8,639 | 1,573 | 1.29 (1.23-1.36) |
| Ethnicity | White | 49,940 | 8,086 | 1.28 (1.25-1.31) |
|  | Non-white | 3,231 | 430 | 1.38 (1.25-1.52) |
| Smoking status | Non-smoking | 25,980 | 4,778 | 1.26 (1.23-1.30) |
|  | Smoking | 27,191 | 3,738 | 1.32 (1.27-1.36) |
| Drinking frequency | ≥3 times/week | 19,491 | 2,998 | 1.26 (1.21-1.31) |
|  | <3 times/week | 33,680 | 5,518 | 1.30 (1.26-1.33) |
| Physical activity | Inadequate | 23,330 | 3,945 | 1.29 (1.25-1.33) |
|  | Adequate | 29,841 | 4,571 | 1.28 (1.24-1.32) |
| Sleep duration | 7-8 hours/day | 35,436 | 5,329 | 1.28 (1.25-1.32) |
|  | <7 or >8 hours/day | 17,735 | 3,187 | 1.29 (1.24-1.33) |
| **TyG-BRI** |  |  |  |  |
| Sex | Female | 34,939 | 4,600 | 1.30 (1.27-1.33) |
|  | Male | 18,232 | 3,916 | 1.31 (1.27-1.36) |
| Age (years) | ≥60 | 18,544 | 4,575 | 1.25 (1.22-1.29) |
|  | <60 | 34,627 | 3,941 | 1.35 (1.32-1.39) |
| Townsend deprivation index | <median | 26,570 | 3,936 | 1.30 (1.26-1.34) |
|  | ≥median | 26,601 | 4,580 | 1.32 (1.28-1.35) |
| Educational level | University or college | 15,988 | 2,060 | 1.34 (1.29-1.40) |
|  | Others | 37,183 | 6,456 | 1.30 (1.27-1.33) |
| Employed status | Employed | 44,532 | 6,943 | 1.31 (1.28-1.34) |
|  | Not employed | 8,639 | 1,573 | 1.29 (1.24-1.34) |
| Ethnicity | White | 49,940 | 8,086 | 1.30 (1.28-1.33) |
|  | Non-white | 3,231 | 430 | 1.43 (1.31-1.55) |
| Smoking status | Non-smoking | 25,980 | 4,778 | 1.27 (1.24-1.31) |
|  | Smoking | 27,191 | 3,738 | 1.36 (1.32-1.40) |
| Drinking frequency | ≥3 times/week | 19,491 | 2,998 | 1.31 (1.26-1.36) |
|  | <3 times/week | 33,680 | 5,518 | 1.31 (1.28-1.34) |
| Physical activity | Inadequate | 23,330 | 3,945 | 1.29 (1.26-1.33) |
|  | Adequate | 29,841 | 4,571 | 1.33 (1.29-1.37) |
| Sleep duration | 7-8 hours/day | 35,436 | 5,329 | 1.33 (1.30-1.37) |
|  | <7 or >8 hours/day | 17,735 | 3,187 | 1.27 (1.24-1.31) |
| **TyG-ABSI** |  |  |  |  |
| Sex | Female | 34,939 | 4,600 | 1.27 (1.23-1.31) |
|  | Male | 18,232 | 3,916 | 1.20 (1.16-1.25) |
| Age (years) | ≥60 | 18,544 | 4,575 | 1.16 (1.12-1.20) |
|  | <60 | 34,627 | 3,941 | 1.32 (1.28-1.37) |
| Townsend deprivation index | <median | 26,570 | 3,936 | 1.20 (1.15-1.24) |
|  | ≥median | 26,601 | 4,580 | 1.28 (1.24-1.32) |
| Educational level | University or college | 15,988 | 2,060 | 1.25 (1.19-1.31) |
|  | Others | 37,183 | 6,456 | 1.24 (1.20-1.27) |
| Employed status | Employed | 44,532 | 6,943 | 1.24 (1.20-1.27) |
|  | Not employed | 8,639 | 1,573 | 1.25 (1.19-1.32) |
| Ethnicity | White | 49,940 | 8,086 | 1.24 (1.21-1.27) |
|  | Non-white | 3,231 | 430 | 1.28 (1.16-1.41) |
| Smoking status | Non-smoking | 25,980 | 4,778 | 1.23 (1.19-1.27) |
|  | Smoking | 27,191 | 3,738 | 1.26 (1.21-1.31) |
| Drinking frequency | ≥3 times/week | 19,491 | 2,998 | 1.23 (1.18-1.28) |
|  | <3 times/week | 33,680 | 5,518 | 1.25 (1.21-1.29) |
| Physical activity | Inadequate | 23,330 | 3,945 | 1.24 (1.20-1.28) |
|  | Adequate | 29,841 | 4,571 | 1.24 (1.20-1.29) |
| Sleep duration | 7-8 hours/day | 35,436 | 5,329 | 1.24 (1.20-1.28) |
|  | <7 or >8 hours/day | 17,735 | 3,187 | 1.25 (1.20-1.30) |

*Note*: Models were adjusted for age, sex, race, employment status, educational level, Townsend deprivation index, smoking status, drinking frequency, sleep duration, and physical activity (excluding stratification variables). Abbreviations: CVD, cardiovascular disease; HR, hazard ratio; CI, confidence interval; SD, standard deviation; TyG, triglyceride-glucose; BMI, body mass index; WC, waist circumference; WHtR, waist-to-height ratio; WWI, weight-adjusted waist index; BRI, body roundness index; ABSI, a body shape index.

**Table S16. Baseline characteristics of participants with complete TyG-related indices and those excluded because of missing TyG-related indices among participants with depression and without baseline CVD.**

| **Variables** | **Included** | **Excluded due to missing TyG-related indices** | ***P*** | **SMD** |
| --- | --- | --- | --- | --- |
| n | 56745 | 10641 |  |  |
| **Sex** |  |  |  |  |
| Male | 19452 (34.3) | 3297 (31.0) | <0.001 | 0.07 |
| Female | 37293 (65.7) | 7344 (69.0) |  |  |
| **Age (mean (SD))** | 55.2 (7.9) | 55.1 (7.8) | 0.197 | 0.014 |
| **Race** |  |  |  |  |
| White | 52708 (92.9) | 9693 (91.1) | <0.001 | 0.07 |
| Non-white | 3790 (6.7) | 869 (8.2) |  |  |
| Missing | 247 (0.4) | 79 (0.7) |  |  |
| **Educational level** |  |  |  |  |
| University or college | 16395 (28.9) | 2966 (27.9) | <0.001 | 0.259 |
| Others | 39640 (69.9) | 7029 (66.1) |  |  |
| Missing | 710 (1.3) | 646 (6.1) |  |  |
| **Employed status** |  |  |  |  |
| Employed | 46729 (82.3) | 8522 (80.1) | <0.001 | 0.065 |
| Not employed | 9703 (17.1) | 2025 (19.0) |  |  |
| Missing | 313 (0.6) | 94 (0.9) |  |  |
| **TDI (mean (SD))** | -0.74 (3.34) | -0.53 (3.43) | <0.001 | 0.063 |
| **Smoking** |  |  |  |  |
| Never | 28846 (50.8) | 5494 (51.6) | 0.001 | 0.041 |
| Ever | 18749 (33.0) | 3418 (32.1) |  |  |
| Current | 8886 (15.7) | 1650 (15.5) |  |  |
| Missing | 264 (0.5) | 79 (0.7) |  |  |
| **Drinking** |  |  |  |  |
| Never | 7068 (12.5) | 1438 (13.5) | <0.001 | 0.052 |
| Special occasions only | 8837 (15.6) | 1723 (16.2) |  |  |
| 1-3 times/month | 7006 (12.3) | 1273 (12.0) |  |  |
| 1-2 times/week | 13390 (23.6) | 2431 (22.8) |  |  |
| 3-4 times/week | 10182 (17.9) | 1878 (17.6) |  |  |
| Daily or almost daily | 10121 (17.8) | 1851 (17.4) |  |  |
| Missing | 141 (0.2) | 47 (0.4) |  |  |
| **Physical activity** |  |  |  |  |
| Adequate | 30768 (54.2) | 5710 (53.7) | 0.004 | 0.034 |
| Inadequate | 24105 (42.5) | 4512 (42.4) |  |  |
| Missing | 1872 (3.3) | 419 (3.9) |  |  |
| **Sleep duration** |  |  |  |  |
| 7-8 hours/day | 37098 (65.4) | 6878 (64.6) | 0.017 | 0.029 |
| <7 or >8 hours/day | 18913 (33.3) | 3591 (33.7) |  |  |
| Missing | 734 (1.3) | 172 (1.6) |  |  |

Note: Values are presented as mean (SD) for continuous variables and n (%) for categorical variables. P values were calculated using t-tests for continuous variables and chi-square tests for categorical variables. TyG, triglyceride-glucose; CVD, cardiovascular disease; TDI, Townsend deprivation index; SMD, Standardized mean differences.


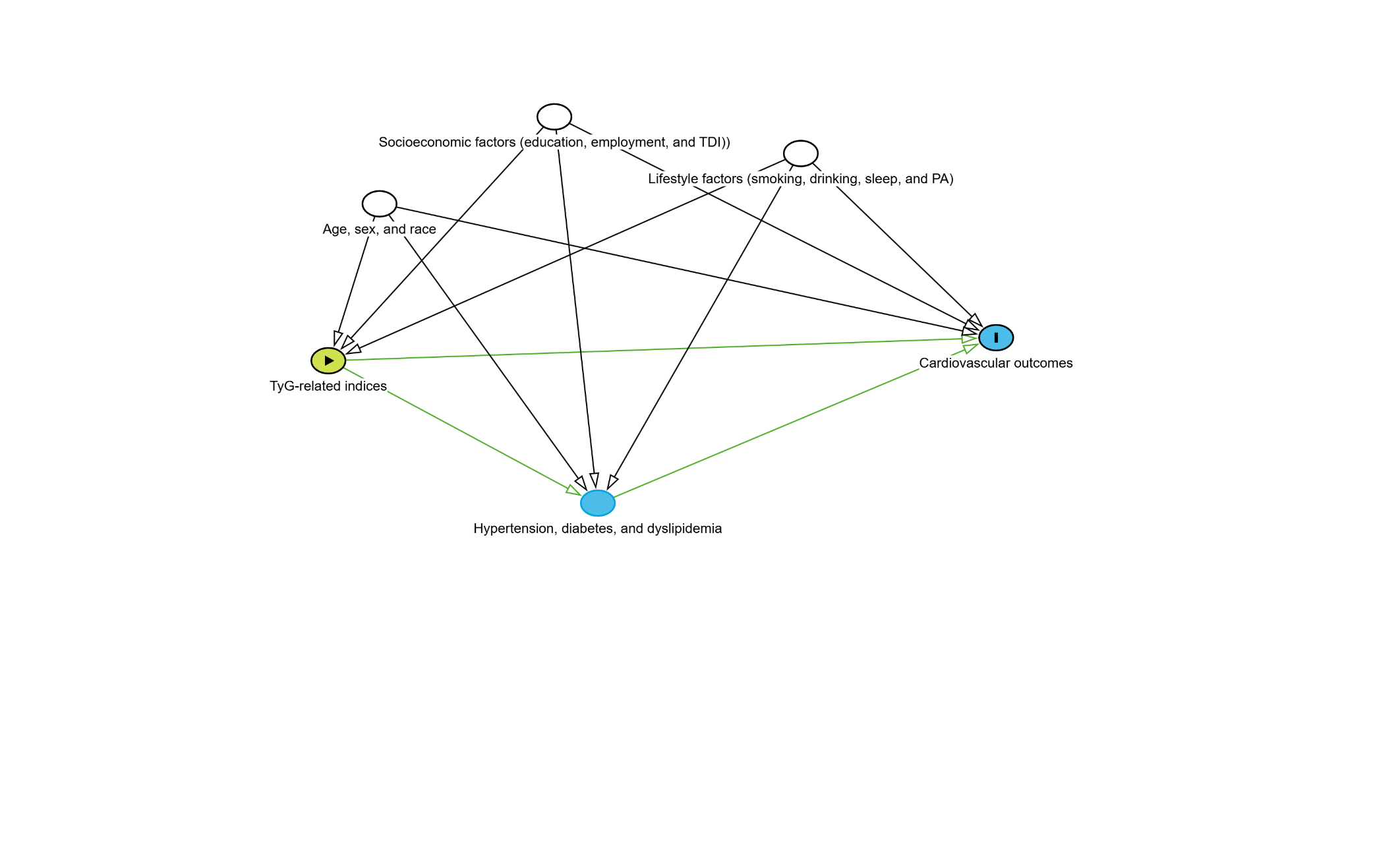


**Figure S1. Directed acyclic graph illustrating the adjusted framework for the association between TyG-related indices and cardiovascular outcomes.**

*Note:* TyG, triglyceride-glucose; TDI, Townsend Deprivation Index; PA, physical activity. Black arrows indicate assumed confounding pathways from upstream demographic, socioeconomic, and lifestyle factors to TyG-related indices, intermediate cardiometabolic conditions, and cardiovascular outcomes. Green arrows indicate the hypothesized total and indirect pathways from TyG-related indices to cardiovascular outcomes, including pathways mediated through hypertension, diabetes, and dyslipidemia. In the primary analysis, demographic, socioeconomic, and lifestyle factors were adjusted for as upstream confounders, whereas hypertension, diabetes, and dyslipidemia were not included in the main adjustment model because they may represent downstream cardiometabolic intermediates. Additional adjustment for baseline hypertension and diabetes was performed as a conservative sensitivity analysis.

**
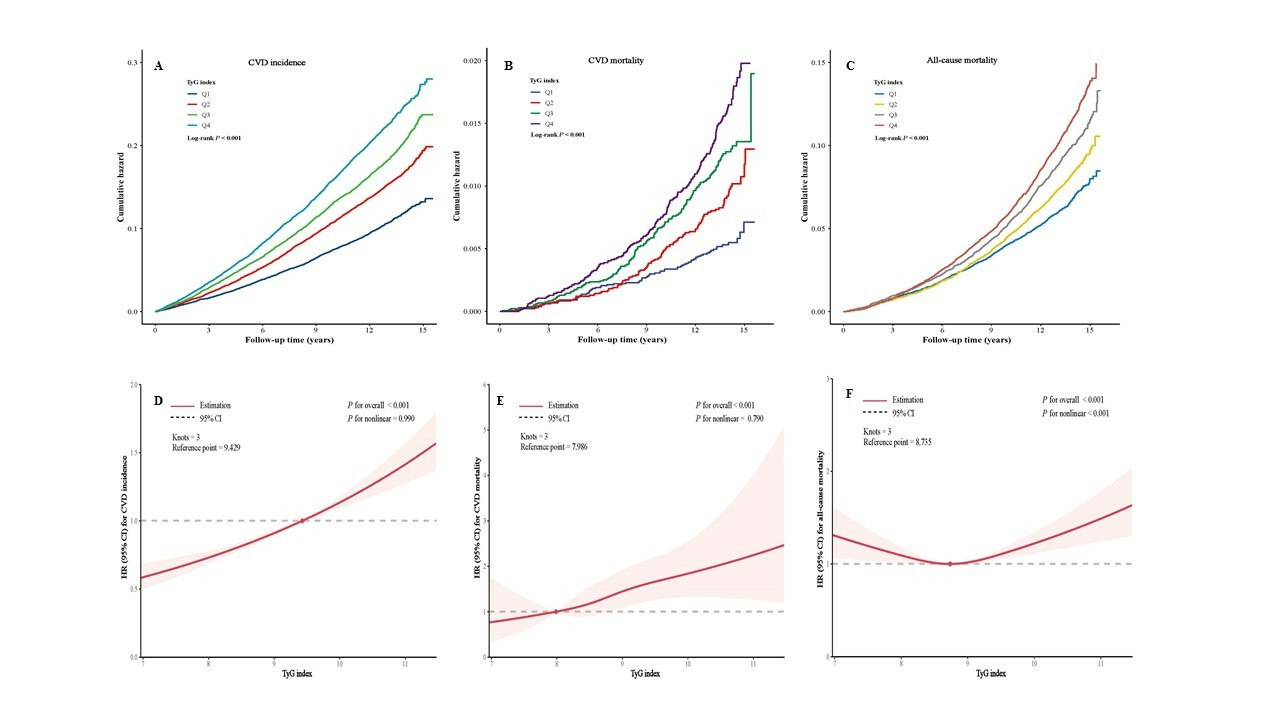
**

**Figure S2. Kaplan-Meier curves of study outcomes according to TyG index quartiles** **(A-C), and dose-response relationship of the TyG index with outcomes by RCS analysis in participants with depression (D-F).**

*Note*: RCS analysis was derived from Cox proportional hazards models adjusted for age, sex, race, employment status, educational level, Townsend deprivation index, smoking status, drinking frequency, sleep duration, and physical activity. A: CVD incidence; B: CVD mortality; and C: All-cause mortality. D: CVD incidence; E: CVD mortality; and F: All-cause mortality.

*Abbreviation*: Q, Quartile; HR, hazard ratio; CI, confidence interval; CVD, cardiovascular disease; TyG, triglyceride-glucose.

**
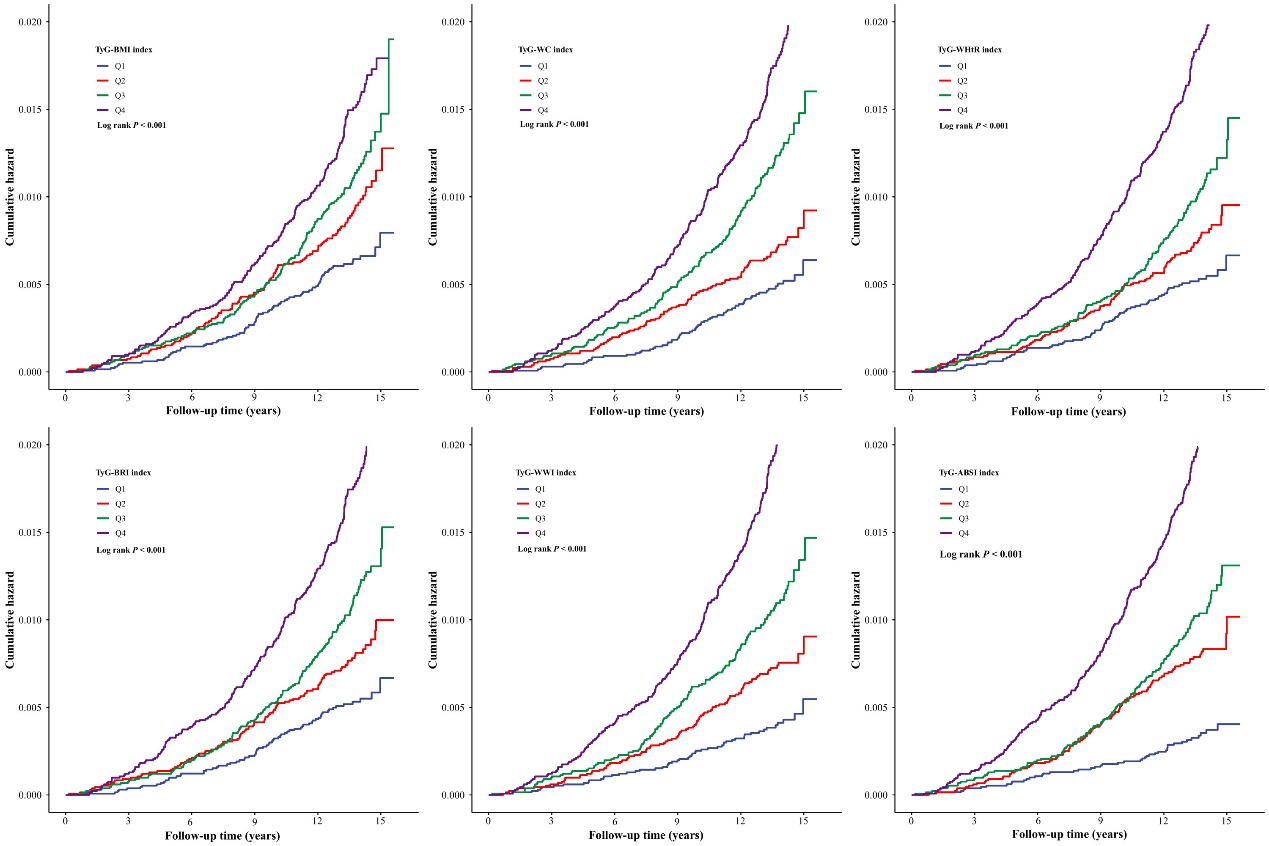
**

**Figure S3. Kaplan-Meier curves of CVD mortality according to the quartiles of TyG-related indices in participants with depression.**

Abbreviation: Q, Quartile; HR, hazard ratio; CI, confidence interval; CVD, cardiovascular disease; TyG, triglyceride-glucose; BMI, body mass index; WC, waist circumference; WHtR, waist-to-height ratio; BRI, body roundness index; ABSI, a body shape index; WWI, weight-adjusted waist index.


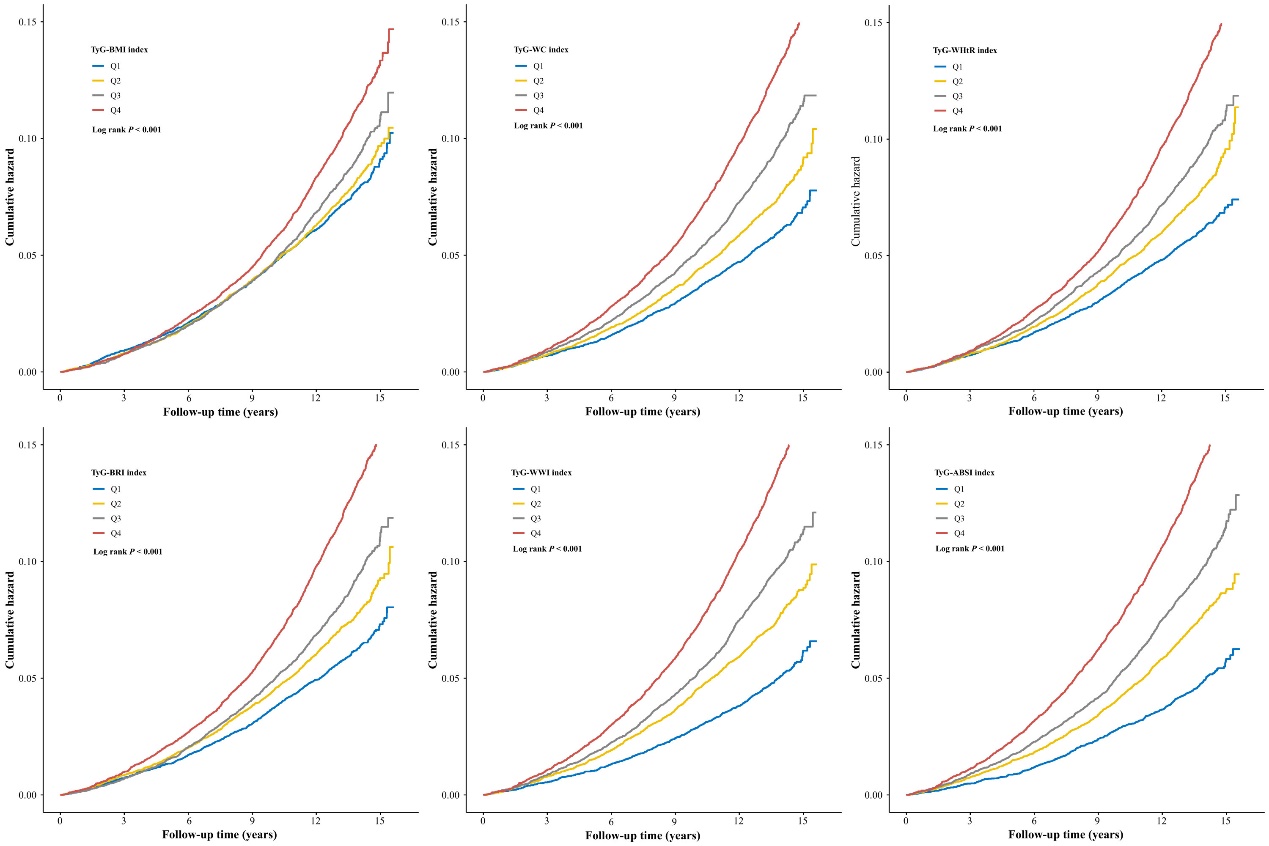


**Figure S4. Kaplan-Meier curves of all-cause mortality according to the quartiles of TyG-related indices in participants with depression.**

Abbreviation: Q, Quartile; HR, hazard ratio; CI, confidence interval; CVD, cardiovascular disease; TyG, triglyceride-glucose; BMI, body mass index; WC, waist circumference; WHtR, waist-to-height ratio; BRI, body roundness index; ABSI, a body shape index; WWI, weight-adjusted waist index.

**
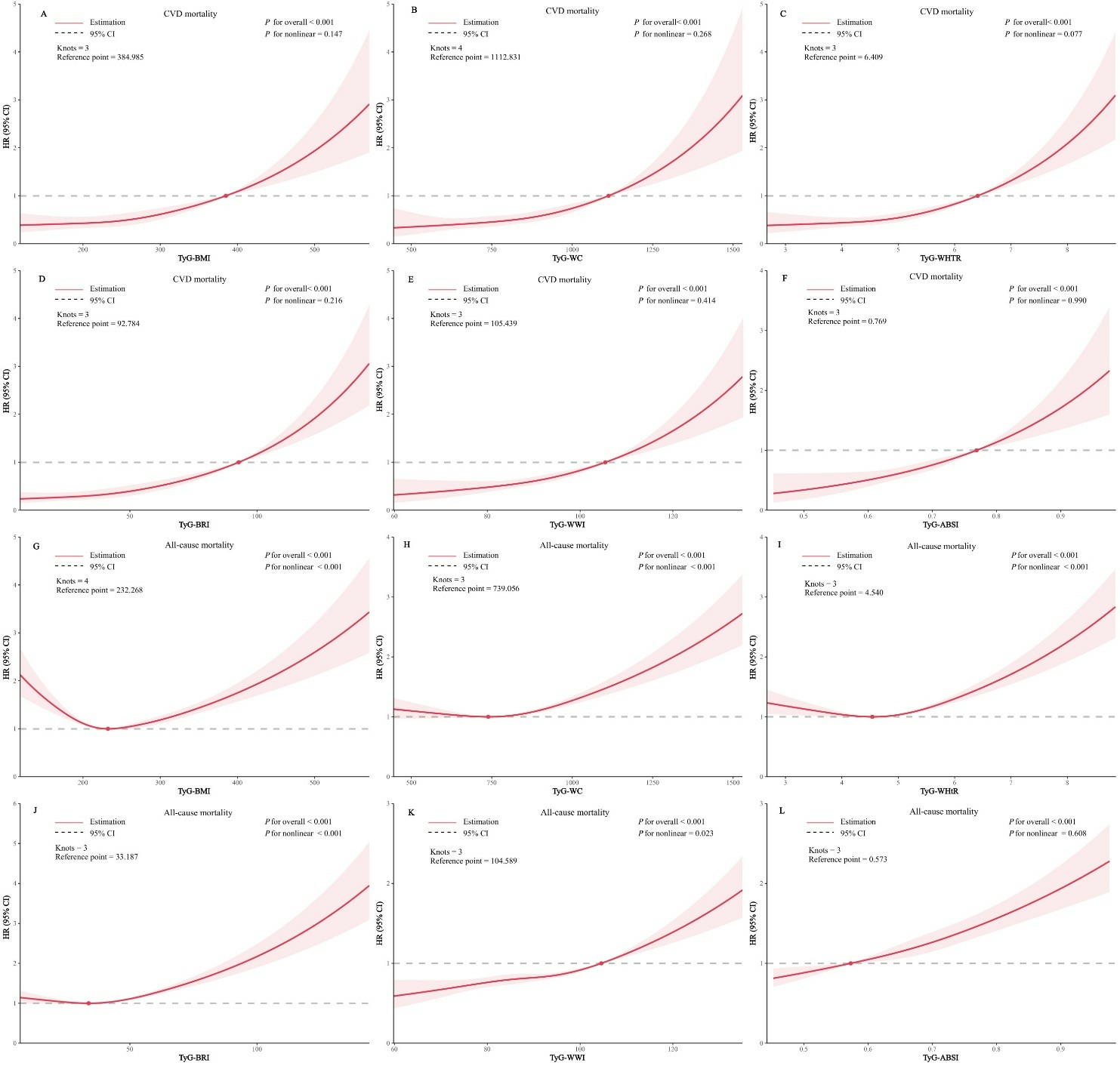
**

**Figure S5. Dose-response relationship of TyG-related indices with CVD and all-cause mortality by RCS analysis in participants with depression.**

*Note*: RCS analysis was derived from Cox proportional hazards models adjusted for age, sex, race, employment status, educational level, Townsend deprivation index, smoking status, drinking frequency, sleep duration, and physical activity. A-F for CVD mortality. A: TyG-BMI index; B: TyG-WC index; C: TyG-WHtR index; D: TyG-BRI index; E: TyG-WWI index; and F: TyG-ABSI index. G-L for all-cause mortality. G: TyG-BMI index; H: TyG-WC index; I: TyG-WHtR index; J: TyG-BRI index; K: TyG-WWI index; and L: TyG-ABSI index.

*Abbreviation*: HR, hazard ratio; CI, confidence interval; CVD, cardiovascular disease; RCS, restricted cubic spline; TyG, triglyceride-glucose; BMI, body mass index; WC, waist circumference; WHtR, waist-to-height ratio; BRI, body roundness index; ABSI, a body shape index; WWI, weight-adjusted waist index.


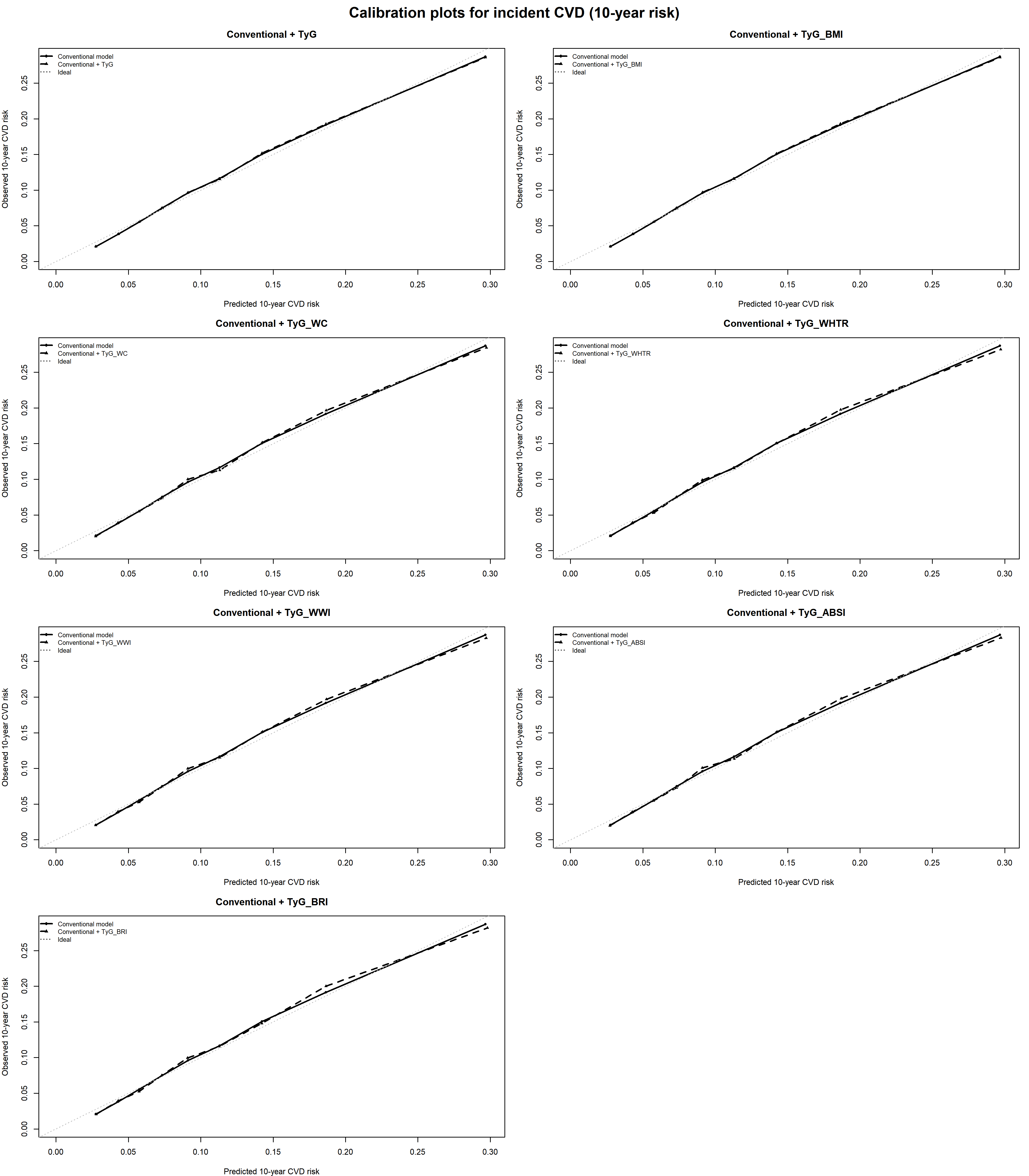


**Figure S6**. **Calibration plots for 10-year incident CVD risk**

Note: Calibration plots comparing predicted and observed 10-year incident CVD risks for the conventional cardiovascular risk model and models additionally including each TyG-related index. The dotted diagonal line represents ideal calibration. The calibration curves for the conventional and extended models were largely overlapping and close to the ideal line, suggesting generally good calibration and no material improvement in calibration after adding TyG-related indices.
